# A hybrid framework for compartmental models enabling simulation-based inference

**DOI:** 10.1007/s00285-026-02409-y

**Published:** 2026-05-27

**Authors:** Domenic P. J. Germano, Alexander E. Zarebski, Sophie Hautphenne, Robert Moss, Jennifer A. Flegg, Mark B. Flegg

**Affiliations:** 1 The School of Mathematics and Statistics, The University of Melbourne, Parkville, VIC Australia; 2 The School of Mathematics and Statistics, The University of Sydney, Camperdown, NSW Australia; 3 Pandemic Sciences Institute, University of Oxford, Oxford, United Kingdom; 4 Melbourne School of Population and Global Health, The University of Melbourne, Parkville, Vic Australia; 5 School of Mathematics, Monash University, Clayton, VIC Australia

**Keywords:** Hybrid simulation, Stochastic modelling, Viral clearance, Extinction, Compartmental modelling, 92Bxx, 37M05

## Abstract

Multi-scale systems often exhibit a combination of stochastic and deterministic dynamics. In compartmental models, low occupancy compartments tend to exhibit stochastic dynamics while high occupancy compartments tend to follow deterministic dynamics. Representing both dynamics with existing methods is challenging. Failing to account for stochasticity in small populations can produce “atto-foxes”, for example in the Lotka-Volterra ordinary differential equation (ODE) model. This limitation becomes problematic when studying the extinction of species or the clearance of infection, but it can be overcome by using discrete stochastic models, such as continuous-time Markov chains (CTMCs). Unfortunately, simulating CTMCs is impractical for many realistic models, where discrete events have very high frequencies. In this work, we develop a mathematical framework to couple continuous ODEs and discrete CTMCs: “Jump-Switch-Flow” (JSF). In this framework, compartments can reach extinct states (“absorbing states”), thereby resolving atto-fox-type problems. JSF has the desired behaviours of exact CTMC simulation, but is substantially computationally faster than existing alternatives, by at least one order of magnitude, and can even obtain constant scaling, irrespective of compartment occupancy. We demonstrate JSF’s utility for simulation-based inference, particularly multi-scale problems, with several case-studies. In a simulation study, we demonstrate how JSF can enable a more nuanced analysis of the efficacy of public health interventions. We also carry out a novel analysis of longitudinal within-host data from SARS-CoV-2 infections to quantify the timing of viral clearance. In this work, we show how JSF offers a novel approach to compartmental model simulation.

**Supplementary Information:**

The online version contains supplementary material available at 10.1007/s00285-026-02409-y.

## Introduction

Dynamical systems are a powerful method for describing the world, and have successfully been applied in many fields. However, modelling populations which change in size across multiple scales remains a challenge Fowler (2021). Population sizes in biological processes often change over orders of magnitude, e.g. the spread of infectious disease (Anderson and May 1991); the boom-and-bust of insect populations (Ludwig et al. 1978); and the immune response to infections, such as HIV (Perelson 2002), and influenza virus (Baccam et al. 2006).

The dynamics of small populations can be heavily influenced by stochastic effects, while for larger populations these fluctuations often average out, justifying the use of continuum models. When the size of the population under consideration does not vary across many orders of magnitude^3.5^, there is usually a natural (and obvious) choice between a stochastic or deterministic model. However, for multi-scale models, this decision remains a challenge, and developing modelling methods that can bridge these scales has been a long-standing goal of applied mathematics (Cotter and Erban 2016; Flegg et al. 2014; Isaacson 2013).

Compartmental models describe how quantities (e.g. the number of molecule, cells, or people) change in a dynamical system. Two popular ways to represent compartmental models are ordinary differential equations (ODEs) and continuous-time Markov chains (CTMCs). Many ODEs one encounters are actually *ensemble averages* of CTMCs, although the mathematical justification of this is not always straightforward (Kurtz 1970, 1971, 1972).

In ODEs, the state of the system changes continuously, which can lead to “atto-fox problems” where populations shrink to infeasibly small sizes, i.e. where a real population would likely have gone extinct (Fowler 2021; Mollison 1991; Lobry and Sari 2015). In CTMCs, the discrete state changes stochastically and there may be absorbing states, e.g. extinction of a population. As a result, CTMCs offer a more natural description of small populations sizes (Bretó et al. 2009), however simulating large populations can be computationally challenging. Moreover, a range of powerful techniques can be brought to bear on ODEs, enabling more thorough analysis. This raises a natural question about what to do when compartment occupancy changes across orders of magnitude.

Exact and approximate algorithms to simulate CTMCs exist Gillespie (1976); Simoni et al. (2019). Exact methods (e.g. Doob-Gillespie, first/next reaction, and rejection based methods) are computationally expensive when state transitions occur at a high rate (Sanft and Othmer 2015). Approximate methods (e.g. Tau-leaping (Gillespie 2001; Cao et al. 2006) and the use of chemical Langevin equations (Rao and Arkin 2003; Gillespie 2000; Gibson and Bruck 2000)) scale to high transition rates but can have unacceptable approximation errors. To overcome the limitations of classical approximate methods, over the past two decades, there has been a concerted effort to develop *hybrid* stochastic-deterministic approaches (Simoni et al. 2019; Kreger et al. 2021; Bressloff and Newby 2014; Rebuli et al. 2017).

Some hybrid modelling approaches involve partitioning the transitions in a CTMC into fast and slow transitions. Despite having significant implementation bookkeeping and overheads, these approaches can be sampled individually and subsequently synchronised in an efficient manner Simoni et al. (2019). Jump-diffusion differential equations partition the model compartments into *fluid* and *discrete* compartments, to construct *hybrid switching jump diffusion* processes Buckwar and Riedler (2011), Angius et al. (2015). Other hybrid simulation techniques have been developed in the context of within-host viral infection Kreger et al. (2021). However, this implementation is not generalised, but tailored to a single system, and does not allow switching between stochastic and deterministic regimes. In the context of determining the hybridisation between stochastic and deterministic couplings, path-integrals have been utilised, Bressloff and Newby (2014), which permit analysis of hybrid processes. Recently, Kynaston et al. (2023) introduced the *regime conversion method* (RCM) considering extended systems that represent each compartment with both a continuous and a discrete version, allowing for the conversion between them Kynaston et al. (2023). In the RCM, switching between the discrete and continuous representation requires a *conversion reaction* that is based on the species occupancy Kynaston et al. (2023). In the work we present here, switching between the discrete and continuous regimes only occurs when the species occupancy crosses a predefined threshold.^3.6^

The hybrid processes we introduce here belong to the class of *piecewise deterministic Markov processes* (PDMPs), originally defined by Davis (Davis 1984). PDMPs have been studied as a general class of stochastic processes (Riedler 2013; Azaïs et al. 2014; Saporta et al. 2016). In this work we consider their use in the context of compartmental models and their role in “plug and play” inference (Bretó et al. 2009).^2.1^

Essential for the real-world utility of many mathematical models is their ability to be calibrated to data. For deterministic compartment models, standard parameter inference methods, such as maximum likelihood and posterior sampling via Markov chain Monte Carlo (MCMC), can readily be applied. For stochastic models these inference problems are usually much harder. Simulation-based inference methods have emerged as a way to handle this challenge, e.g. the *particle filter* (Arulampalam et al. 2002; Kitagawa 1996) and *approximate Bayesian computation* (Alahmadi et al. 2020) in the Bayesian setting, and *iterated filtering* (Ionides et al. 2006; Bretó et al. 2009) in the frequentist setting.^1.1^

Calibrated multi-scale simulations of ODE (compartment) models is an important milestone towards accurate predictive models that address stochastic behaviour. In a recent paper, Trindade and Zygalakis develop a hybrid scheme for chemical kinetics over different scales with the goal of demonstrating the utility for parameter estimation  (Trindade and Zygalakis 2024). In this paper, a CTMC is used to model reaction events on small populations (below a threshold $I_1>0$) and for better efficiency Tau-leaping is used for reactions that only impact large populations (above a threshold $I_2>I_1$). Between these two thresholds, a “blending function” is used to create a linear transition between an accurate CTMC and efficient Tau-leaping treatment of the reactions. The hybrid adoption of CTMC and Tau-leaping regimes has the property of maintaining appropriate levels of noise at large populations but struggles as populations grow much larger where a Chemical Langevin or deterministic ODE description may be more computationally appropriate.

In this paper, we present a simple and efficient hybrid simulation method which we call *Jump-Switch-Flow* (JSF). This method enables adaptive transitions between stochastic and deterministic regimes across compartments, while ensuring conservation principles are maintained. In this approach, compartments can individually switch regimes, allowing for the compartments to be split into stochastic and deterministic subsets, while remaining coupled via a *jump clock* that utilises the fact that the deterministic compartments result in the jump times forming an *inhomogeneous* Poisson process^3.11^. This regime switching presents unique challenges for matching at interfaces: ensuring that the deterministic regime represents the expected state after transition between regimes and that mass is conserved. Conservation is not always essential when it pertains to small population sizes, however in many cases sustained small populations have a significant impact on larger populations (e.g. enzymes in chemical processes and viruses within hosts). We demonstrate the utility and properties of the JSF method through simulation studies and a case study in which we reanalyse existing longitudinal SARS-CoV-2 viral load datasets.

Two simulation studies are presented to explore the properties of JSF and demonstrate its use in an inference setting. In the first simulation study, we compare the computational efficiency and accuracy of JSF with the exact Doob-Gillespie method and the Tau-leaping method. In the second simulation study, we demonstrate the types of insight available when using an approach that supports absorbing (extinction) states.

In the case study, we demonstrate the significance of being able to perform inference with models with absorbing states (i.e. models in which one of the compartments can go extinct.) We reanalyse longitudinal SARS-CoV-2 viral load data (Ke et al. 2022) using JSF to simulate a TEIRV (Target cells – Eclipsed cell – Infectious cells – Refractory cell – Virions) model and infer the state of host and virus across the infection. Understanding how a viral infection is cleared has important ramifications for both treatment and prevention. For example, the initial exposure may fail to initiate a systematic infection, and understanding the conditions under which this happens is important for infection prevention Pearson et al. (2011). Moreover, the infection may reach low levels, potentially escaping detection, but the virus may rebound and cause disease. Understanding when the virus has been cleared is important for optimising treatment duration. Inferring viral clearance can be computationally challenging (Yan et al. 2016), and this case study demonstrates how JSF enables the tractable estimation of viral clearance.

## Methods

### Jump-Switch-Flow mathematical framework

Consider a compartmental model with *n* compartments, $\vec {V} = \left( v_1, ..., v_n\right)$, where the state variable, $v_i := v_{i}(t)$, represents the value of the $i$th compartment at time $t$. For example, $v_i$ could be the number of people infected with a pathogen, or the quantity^3.8^ of a molecule in a cell. The state variables $v_i$ may take values from different domains, depending on the resolution needed, for example in an ODE, $v_i$ will have real values, and in a CTMC, $v_i$ might have integer values.

When the variables represent quantities, typically, discrete values are used to represent small populations, while larger populations are represented with a continuum. We define the *switching threshold parameter*, $\Omega _i\in \mathbb {Z}_{\ge 0}$, as the value where the *i*th compartment transitions between discrete and continuous dynamics. If a compartment $v_{i} \in \{0,1,\ldots ,\Omega _{i}\}$, we call it *discrete* (or *jumping*), and if $v_{i} \in (\Omega _{i},\infty )$, we call it *continuous* (or *flowing*). To accommodate both scales, let the domain of $v_{i}$ be the set $\textbf{E}_{i} = \{0,1,\ldots ,\Omega _{i}\}\cup (\Omega _{i},\infty )$ (with the $\sigma$-algebra generated by the singletons of $\{0,1,\ldots ,\Omega _i\}$ and the open intervals of $(\Omega _i,\infty )$).^2.1^ The state space for our process is $\textbf{E} = \textbf{E}_1 \times \ldots \times \textbf{E}_{n}$, with the product $\sigma$-algebra.^2.1^ While the switching threshold can be compartment specific, for ease of exposition, we will only consider a single threshold shared between all compartments, i.e. $\Omega _i = \Omega$.

At each time *t*, the components of $\vec {V}$ can be partitioned into $\vec {V}_F$ and $\vec {V}_J$, where $\vec {V}_F$ contains the flowing variables $v_i > \Omega$, and $\vec {V}_J$ contains the jumping variables $v_i \le \Omega$. Therefore, at any moment in time $\dim {(\vec {V}_F)}$ of the *n* compartments are flowing, and $\dim {(\vec {V}_J)}$ of the *n* compartments are jumping, with $\dim {(\vec {V}_F)} + \dim {(\vec {V}_J)} = n$.

The dynamics of each compartment $v_i$ are described by a set of *m* reactions $\mathcal {R} = \left\{ {r}_k \right\} _{k=1}^m$. Each reaction ${r}_k$ is defined by two properties: the rate (per unit time) at which it occurs, $\lambda _{k}$, which (usually) is a function of the state $\vec {V}$; and the reaction’s effect on the state, i.e. the change $\eta _{ik}$ to the size of compartment $v_i$ when reaction ${r}_k$ occurs. As a vector, $\vec {\lambda } \in \mathbb {R}^{m}$ is referred to as the *propensity vector*. As a matrix, $\eta \in \mathbb {Z}^{n,m}$ is referred to as the *stoichiometric matrix*. For ODE models that only contain flowing variables, these reactions occur continuously and are written in the form:

1 $$\begin{aligned} \frac{\textrm{d}\vec {V}}{\textrm{d}t} = \eta \vec {\lambda }(t,\vec {V}). \end{aligned}$$

For CTMC models, reactions in the system $\mathcal {R}$ occur as discrete events. In the latter case, each reaction ${r}_k$ has a separate propensity described by $\lambda _k(t,\vec {V})$. This propensity remains constant between reactions, but when a reaction ${r}_k$ occurs, there is a change in $\vec {V}$ (as specified by the elements of $\eta _{\cdot k}$), and therefore in $\vec {\lambda }(t,\vec {V})$.

As an example, consider the SIR model of epidemics, which, in ordinary differential equations, has the form $dS/dt=-\beta S I/(S + I + R)$, $dI/dt=\beta S I/(S + I + R) - \gamma I$, and $dR/dt = \gamma I$. The state of this model is $\vec {V} = (S,I,R)^\intercal$, and there are two “reactions”: infections (${r}_1$) and recoveries (${r}_{2}$). For infections, the rate of reaction may be modelled by $\lambda _1=\beta S I/(S+I+R)$, and the entries of the associated column of the stoichiometric matrix are $\eta _{1,1} = -1$, $\eta _{2,1} = 1$ and $\eta _{3,1}=0$. For recoveries, the rate of reaction may be modelled by $\lambda _2=\gamma I$, and the entries of the associated column of the stoichiometric matrix are, $\eta _{1,2}=0$, $\eta _{2,2} = -1$, and $\eta _{3,2} = 1$. Therefore, written in product form, the system is:^3.9^

2 $$\begin{aligned} \frac{\textrm{d}}{\textrm{d}t} \begin{pmatrix} S\\ I\\ R \end{pmatrix} = \begin{pmatrix} -1 & 0\\ 1 & -1\\ 0 & 1 \end{pmatrix} \begin{pmatrix} \frac{\beta S I}{S + I + R}\\ \gamma I \end{pmatrix}. \end{aligned}$$

We define $\mathcal {R}_J\subseteq \mathcal {R}$ as the subset of reactions treated as stochastic *jump* events. The set $\mathcal {R}_J$ can be defined more precisely in two ways. While we use only the second in this manuscript, both are described for clarity. The first way to define $\mathcal {R}_J$ is as: $\mathcal {R}_J = \left\{ {r}_k:\exists i \text { s.t. } v_i\in \vec {V}_J \text { and } \eta _{ik}\ne 0 \right\}$. In this definition, a reaction ${r}_k$ is included in $\mathcal {R}_J$ if and only if the reaction has some material effect on a jumping (discrete) compartment $v_i\in \vec {V}_J$. This is a minimum requirement; a reaction should not be permitted to evoke a continuous change in a discrete compartment. However, where it makes sense to do so, it is possible to allow reactions to make discrete changes to flowing (continuous) compartments. The second way to define $\mathcal {R}_J$, which we use throughout this manuscript, captures a larger set of reactions:

$$ \mathcal {R}_J = \left\{ {r}_k:\exists i \text { s.t. } v_i\in \vec {V}_J \text { and } \left( \eta _{ik}\ne 0 \text { or } \partial _{v_i}\lambda _k \ne 0\right) \right\} .$$

In this definition, a reaction is included in $\mathcal {R}_J$ if either (1) it causes a material change in jumping (discrete) compartments *or* (2) it is influenced/caused by a discrete compartment (e.g. as catalyst of some reaction).

Reactions in $\mathcal {R}_J$ are simulated using stochastically sampled times, similar to CTMC models (and described below in the section on ‘Jump events’). It is important to note that unlike time homogeneous CTMC models, the propensities are *not* constant because the state $\vec {V}$ (and therefore $\vec {\lambda }$) are continuously varying. When any reaction ${r}_k\in \mathcal {R}_J$ occurs, we say the system has *jumped* and an instantaneous change of $\eta _{ik}$ for each compartment $v_i$ occurs (irrespective of whether $v_i\in \vec {V}_J$ or $v_i\in \vec {V}_F$, to ensure the conservation of mass). We therefore refer to reactions in $\mathcal {R}_J$ as *jumps*. The reactions in $\mathcal {R}_F=\mathcal {R}\setminus \mathcal {R}_J$ represent the continuous change of value of the relevant compartments, all of which are deterministic (see section on ‘Flow events’.) We therefore refer to reactions in $\mathcal {R}_F$ as *flows*. At any moment in time $|\mathcal {R}_F|$ is the number of reactions which are flowing, and $|\mathcal {R}_J|$ is the number of reactions which are jumping, such that $|\mathcal {R}_F| + |\mathcal {R}_J| = m$.

Finally, the hybrid model that we propose is capable of *switching*. Switching events occur when the size of a compartment $v_i$ crosses the threshold $\Omega$ and therefore changes membership between the flowing $\vec {V}_F$ and jumping $\vec {V}_J$ sets (see sections on ‘Jump clock updates’ and ‘Switching events’). Importantly, switching events may also impact which reactions are jumps, $\mathcal {R}_J$. As such, switches are paradigm-defining events which ought to occur infrequently in comparison to jumps (which may occur frequently) and flows (which occur continuously).

Due to the way that $\mathcal {R}$ is partitioned, it is possible to order the rows and columns of $\eta$ into the upper-triangular block form:

3 $$\begin{aligned} \eta = \left( \begin{array}{c|c} \eta _{FF} & \eta _{FJ} \\ \hline 0 & \eta _{JJ} \end{array}\right) , \end{aligned}$$

where $\eta _{FF} \in \mathbb {Z}^{\dim {(\vec {V}_F)} , \vert \mathcal {R}_F \vert }$, $\eta _{JJ} \in \mathbb {Z}^{\dim {(\vec {V}_J)}, \vert \mathcal {R}_J \vert }$ and ${\eta }_{FJ} \in \mathbb {Z}^{\dim {(\vec {V}_F)} , \vert \mathcal {R}_J \vert }$ refer to stoichiometric coefficients for changes in flowing compartments under flows, jumping compartments under jumps, and flowing compartments under jumps, respectively. Note that, the lower left corner is zeros because, by definition, flowing reactions cannot influence the state of jumping variables. Written as a system of equations analogous to (1), the hybrid JSF framework we propose formally takes the following form. For any time interval $t_{i}<t<t_{i+1}$ between switching events *i* and $i+1$:

4 $$\begin{aligned} \frac{\textrm{d} \vec {V}_F}{\textrm{d} t}&= \eta _{FF} \vec {\lambda }_{F}(t,\vec {V}) + {\eta }_{FJ} \vec {\Lambda }_{J}(t,\vec {V}), \end{aligned}$$

5 $$\begin{aligned} \vec {V}_J(t)&= \vec {V}_J(t_{i}) + \eta _{JJ} \int _{t_{i}}^t \vec {\Lambda }_{J}(s,\vec {V}) \ \textrm{d} s, \end{aligned}$$

where $\vec {\lambda }_{F}\in \mathbb {R}^{\vert \mathcal {R}_F \vert }$ are the reaction rates of flows and $\vec {\Lambda }_{J}$ is a stochastic vector of $\vert \mathcal {R}_J \vert$ delta-function spike trains that are derived from the realisations of $\vert \mathcal {R}_J \vert$ different jumps sampled at rates which are dependent on the dynamic changes in the propensities $\vec {\lambda }_{J}\in \mathbb {R}^{\vert \mathcal {R}_J \vert }$ for these jumps (see ‘Jump events’).

**Fig. 1:**
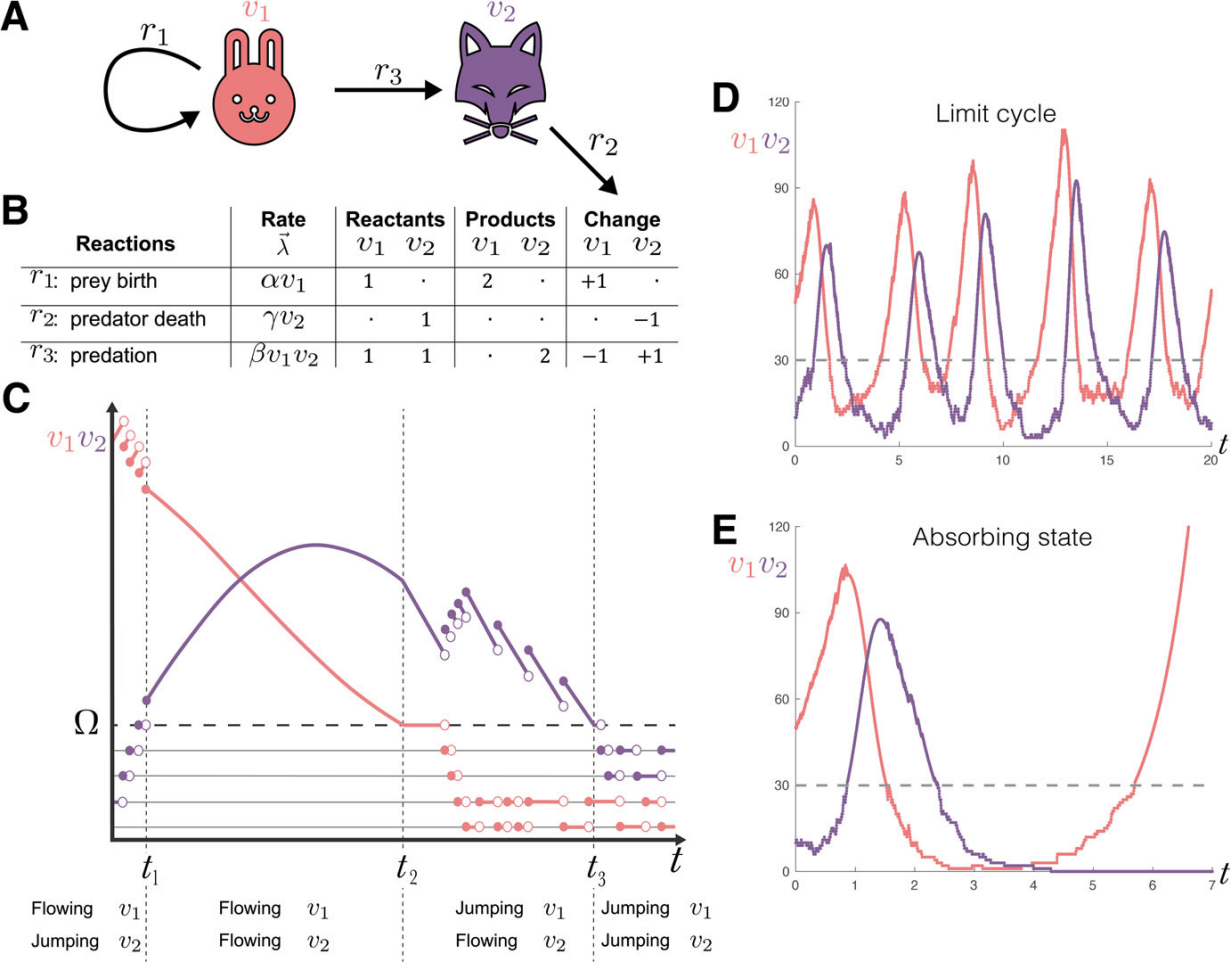
JSF provides a way to capture both the continuous deterministic dynamics of large populations and the discrete stochastic dynamics of small populations. **A** A compartmental model representation of the Lotka-Volterra system (6): prey ($v_1$, depicted as red rabbits which, in reaction $r_1$, reproduce at rate $\alpha$), predators ($v_2$, depicted as purple foxes which, in reaction $r_2$, die at rate $\gamma$), and their interaction (predation of rabbits by foxes, in reaction $r_3$, at a rate proportional to $\beta$). **B** The compartmental model can be formalised as a reaction network via a stoichiometry matrix $\eta$ which details each species role in the reactions. The reactants consumed $\eta ^-$ and the products produced $\eta ^+$ can be written explicitly with $\eta = \eta ^+ - \eta ^-$. **C** A hypothetical JSF trajectory demonstrating how the representation captures the continuous variation of some compartment-reaction pairs (flow) and the discrete stochastic changes (jumps) in others. Transitioning (switching) between a continuous and discrete state occurs at a threshold $\Omega$ which is indicated with a horizontal dashed line. **D** An example trajectory from the Lotka-Volterra model exhibiting the characteristic cyclical behaviour with additional stochasticity influencing the dynamics at low population sizes. **E** An example trajectory showing the possibility for the predator species to go extinct and the subsequent growth of the prey species

### Example: Lotka-Volterra model

The Lotka-Volterra model describes the populations of two species: prey, $v_{1}$, and predators, $v_{2}$. The prey reproduce at rate $\alpha$, the predators die at rate $\gamma$ and new predators are produced through predation at rate $\beta$. When modelled with ODEs, this gives us the following system:

6 $$\begin{aligned} \begin{aligned} \frac{d v_{1}}{dt} = \alpha v_{1} - \beta v_{1}v_{2},\\ \frac{d v_{2}}{dt} = \beta v_{1}v_{2} - \gamma v_{2}. \end{aligned} \end{aligned}$$

The periodic solutions to (6) persist even when the predator species reaches infeasible population sizes: populations of order $10^{-18}$ giving us “atto-foxes”.

Figure 1A shows a compartmental diagram of the Lotka-Volterra model with the reaction: ${r}_1$, birth of $v_1$; ${r}_{2}$, death of $v_2$; and ${r}_{3}$, conversion of $v_1$ into $v_2$. Figure 1B shows a representation of the reactions and their corresponding stoichiometric matrix.

Figure 1C depicts a hypothetical trajectory of the Lotka-Volterra model when represented as a JSF process. There are three switching events (at $t_1$, $t_2$ and $t_3$), and each configuration of discrete (jumping) and continuous (flowing) states are featured:

1. Before $t_1$, the representation is $\vec {V}_F = v_1$ and $\vec {V}_J = v_2$ and the reactions are $\mathcal {R}_J = \left\{ {r}_2, {r}_3 \right\}$ (hybrid regime).
2. During $$[t_1,t_2)$$, the representation is $$\vec {V}_F = (v_1,v_2)^\intercal $$ and $$\vec {V}_J = \varnothing $$ and the reactions are $$\mathcal {R}_J = \varnothing $$ (ODE regime).
3. During $$[t_2,t_3)$$, the representation is $$\vec {V}_F = v_2$$ and $$\vec {V}_J = v_1$$ and the reactions are $$\mathcal {R}_J = \left\{ {r}_1, {r}_3 \right\} $$ (hybrid regime).
4. From $t_3$ onwards the representation is $\vec {V}_F = \varnothing$ and $\vec {V}_J = (v_1,v_2)^\intercal$ and the reactions are $\mathcal {R}_J = \mathcal {R}$ (CTMC regime).

Figures 1D and E show two instances of random trajectories sampled from the Lotka-Volterra process as represented with JSF with a switching threshold of $\Omega = 30$. In the trajectory in Fig. 1D the populations undergo several cycles, while in Figure 1E, with the inclusion of discrete stochastic behaviour, the predator species goes extinct, allowing the prey to grow exponentially.

### Mathematical detail

Here we provide the mathematical details of JSF: the evolution and computation of values for flowing compartments (see ‘Flow events’); the evolution and computation of values for jumping compartments (see ‘Jump events’); the sampling of jump times (see ‘Jump clock updates’); and the switching of compartments between jumping and flowing regimes (see ‘Switching events’). Further details, including pseudo-code listings of the algorithms used to sample from the JSF process, are given in SI Section 1.

We assume that the deterministic ODE admits a unique solution on the time interval of interest. A sufficient condition is that the ODE vector field is locally Lipschitz and that trajectories remain bounded. For the models considered here, this holds due to standard population bounds in the case of the SIRS model, and can easily be verified for the TEIRV and Lotka-Volterra models. The construction of the process and its basic properties, including the associated generator, strong Markov property, and well-posedness under mild Lipschitz and boundedness conditions, are standard and have been treated in general (Davis 1993) and in the context of biochemical jump processes specifically (Anderson and Kurtz 2015). ^2.1^

#### Flow events

Equation (4) contains the differential equations describing the evolution of the flowing compartments. If we consider an interval of time between jumps, $\left[ t_0, t_1\right]$, then $\vec {V}_J(t)$ remains constant and therefore, $\vec {\Lambda }_{J}(t,\vec {V}) = \vec {0}$ across this interval. Therefore, the differential equations for the flowing compartments are:

7 $$\begin{aligned} \frac{\textrm{d} \vec {V}_F}{\textrm{d} t} = \eta _{FF} \vec {\lambda }_{F}(t,\vec {V}). \end{aligned}$$

This is a standard dynamical system of ODEs, which we numerically integrate forward in time over discrete time steps of size $\Delta t$. While an arbitrary order method may be used, we use a simple forward Euler method:

8 $$\begin{aligned} \vec {V}_F(t+\Delta t)=&\vec {V}_F(t) + \Delta \vec {V}_F(t)\end{aligned}$$

9 $$\begin{aligned} =&\vec {V}_F(t) + \Delta t \, \eta _{FF} \vec {\lambda }_{F}(t,\vec {V}) + O(\Delta t^2). \end{aligned}$$

**Fig. 2:**
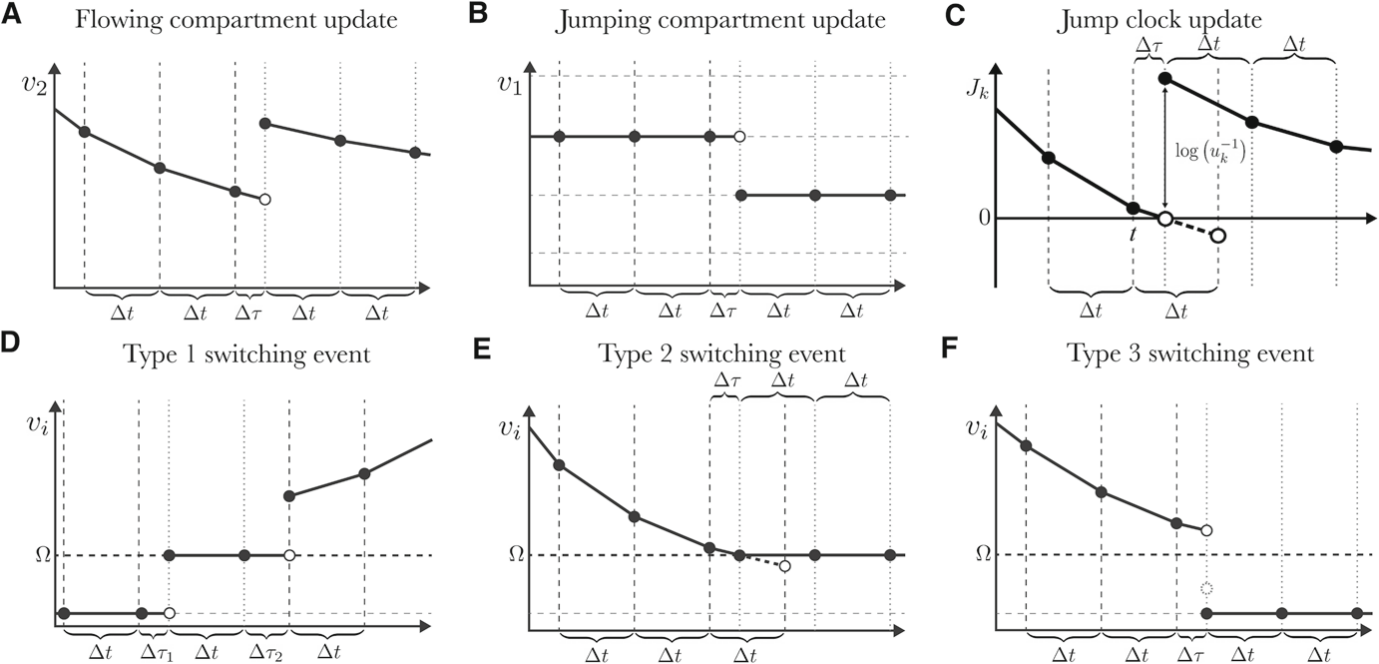
The JSF process can be numerically integrated. **A** and **B**: Dynamic changes in flowing compartments (**A**) and jumping compartments (**B**). Flows are computed using the time step $\Delta t$. If a jump occurs within a flow update, the flows are adjusted up until the corresponding time $\Delta \tau$ as a time step instead of $\Delta t$ (indicated by a discontinuity in both **A** and **B**). Jumps occur when a jump clock reaches zero. **C**: The jump clock $J_k$ for reaction *k* counts down until event *k* occurs when $J_k=0$, i.e. between *t* when $J_k>0$ and $t+\Delta t$ when $J_k<0$. The time of the jump is found by interpolating for $t+\Delta \tau$ using Equation (17), where $\Delta \tau < \Delta t$. Once the jump has occurred and the stoichiometric updates have been applied, $J_k$ is reinitialised by sampling $J_k = -\log (u_k)$ where $u_k\sim \text {Unif}(0,1)$. The clock is then decremented with successive time steps $\Delta t$ until the next jump *k* is realised. **D** A switching event of type 1 occurs when a discrete compartment jumps above the switching threshold, $\Omega$. This is shown here after the jump time step $\Delta \tau _2$. **E** A switching event of type 2 occurs when a flowing compartment reaches the switching threshold, $\Omega$. Here, the time at which the switch occurs is found using continuation. **F** A switching event of type 3 occurs when a flowing compartment is caused to go below the switching threshold, $\Omega$, due to a jump in another compartment. Here the jump has caused the flowing compartment to end at the dotted circle (which we denote as $\hat{v}_i$). This compartment is then re-labelled as jumping. Since jumping compartments require integer states, the state is re-initialised to $\lceil \hat{v}_i \rceil$ with probability $v_i - \lfloor \hat{v}_i \rfloor$ and otherwise to $\lfloor \hat{v}_i \rfloor$. This ensures the expected value of the state is maintained at the dotted circle (in the example presented here, the variable was rounded down). For all switching events, membership of $V_F$ and $V_J$ is recomputed after switching

Figures 2**A** and 2**B** depict examples of a flowing compartment and a jumping compartment, respectively. Flow updates are given at intervals of discrete time step $\Delta t$ as indicated. In the Jump-Switch-Flow algorithm, flowing time steps are bounded above by a finite, but small, $\Delta t$. Flowing time steps have this maximum duration if no jumps are found on this time interval (that is, if (7) remains valid). If a jump occurs at $\Delta \tau < \Delta t$ from any given time (see Section ‘Jump events’) then a flowing time step of $\Delta \tau$ is used instead of $\Delta t$ in Equation (8). In this case, a jump is instantaneously applied after the flow event and the next interval of time is associated with new flow rates $\vec {\lambda }_F$ (which are therefore updated at the exact moment of time of the jump event). We note therefore that the error associated with the ODE solver is determined by the size of the finite time interval size $\Delta t$ according to classical ODE error analysis. In this case, since we use forward Euler, the local truncation error scales as $\mathcal {O}(\Delta t^2)$, while the global truncation error scales as $\mathcal {O}(\Delta t)$, over a finite time horizon. Section SI 1C provides a detailed global truncation error analysis for the birth-death process.^2.2^ Using Equation (8), over each time step (flowing interval), flow events only directly affect flowing compartments as can be seen in Fig. 2**A**. Jumping compartments, such as those shown in Fig. 2**B**, are not directly affected by flowing events because the timing of a jumping event is predetermined according to approximations in the trajectories of the flowing compartments. These approximations have the same accuracy as the ODE solver; for example, we assume a linear changes in the flowing compartments consistent with the forward Euler method used in Equation (8) (see Section “Jump events” for details). As indicated by the discontinuities in Fig. 2**A** and Fig. 2**B**, jump events may be applied to both flowing and jumping compartments in order to conserve mass in a manner which we shall now describe.

#### Jump events

The reactions in $\mathcal {R}_J$ produce discontinuous jumps in the state vector $\vec {V}$. In Equations (4) and (5), each element in the vector $\vec {\Lambda }_{J}$ corresponds to a reaction in $\mathcal {R}_J$. Consider, for example, $r_k \in \mathcal {R}_J$. The $k^{\text {th}}$ element of $\vec {\Lambda }_{J}$ is:

10 $$\begin{aligned} \Lambda _{J}^{(k)} = \sum _{i} \delta \left( t-t_{i}^{(k)} \right) , \end{aligned}$$

where $\delta (\cdot )$ is the Dirac delta function and $t_{i}^{(k)}$ is the time at which the reaction $r_k$ occurs for the *i*th time. In this way, the term $\vec {\Lambda }_{J}$ in Equations (4) and (5) manifests as discrete jumps in both $\vec {V}_F$ and $\vec {V}_J$ at each jump. For the sake of the simulation, the computation of the jump times $t_{i}^{(k)}$ for each instance *i* of each reaction $r_k\in \mathcal {R}_J$ is all that is required. The stoichiometric matrix describes the change to each compartment when that jump event occurs.

The rate of reaction $r_k$, $\lambda _k(t,\vec {V})$, may be state-dependent so can change due to the continuous change caused by flows. Figures 2**A** and 2**B** depict an example of flowing and jumping compartment (respectively) experiencing a jump event. In this illustration, we note that if $\lambda _k$ depends on $v_2$, the event time $t_i^{(k)}$ is not sampled from an exponential distribution which requires a constant propensity/rate between events. Formally, the jump times form an inhomogeneous Poisson process. There are multiple ways to sample the jump times $t_{i}^{(k)}$, see Klein and Roberts (1984) for a detailed discussion. We use a variant of the Next Reaction Method Gibson and Bruck (2000) (which is an optimised variant of the Doob-Gillespie method Gillespie (1976)) to sample jump times. We first note that the propensity for a jump depends only on the instantaneous state $\vec {V}$, and therefore at time $t_0$ if there has been $i-1$ jumps associated with reaction ${r}_k$, it has no bearing on the distribution of the time $t_i^{(k)}$. Therefore, we shall simply denote $t_i^{(k)} = t_k$ as the *next* jump time for reaction ${r}_k$. The cumulative probability function from which $t_k$ is sampled depends on the current time, $t_0$, and the evolution of the state variables in time, $\vec {V}(t)$. The cumulative distribution for the time of the next event associated with $r_k$ is:

11 $$\begin{aligned} \text {CDF}(t;k) = 1 - \exp \left\{ - \int _{t_{0}}^{t} \lambda _{k}(s,\vec {V}(s)) ds \right\} . \end{aligned}$$

To sample $t_k$, inverse transform sampling is used Klein and Roberts (1984), i.e. sample $u_{k}\sim \text {Unif(0,1)}$ and then solve $\text {CDF}(t_k;k) = u_k$ for $t_k$. To account for the varying state, we define a new function, $J_k(t)$ the *jump clock* for reaction ${r}_k$, using Equation (11) and solving $\text {CDF}(t_k;k) = u_k$ (inverse transform sampling). The jump clock acts as a timer, identifying the time $t_k$ for when $r_k$ next occurs, when $J_k(t_k)=0$. The function $J_k$ is defined by:

12 $$\begin{aligned} J_k(t_k) = -\log (u_{k}) - \int _{t_{0}}^{t_k} \lambda _{k}(s,\vec {V}(s)) \ \textrm{d}s, \end{aligned}$$

noting that $u_k$ and $1-u_k$ have the same distribution. In general, we cannot solve directly for $t_k$, so we solve for it numerically by tracking the value of $J_k(t)$ as $\vec {V}$ evolves through flows, jumps and switches.

For each reaction ${r}_k$, at some initial time, for example $t_{i-1}^{(k)}$, being the continuous time of the $(i-1)^{\text {th}}$ jump associated with reaction $r_k$, we sample $u_k$ and initialise $t_0 = t_{i-1}^{(k)}$. The initial value of $J_k(t)$ is therefore equal to the positive number $\log (u_{k}^{-1})$ (since $u_{k}\sim \text {Unif(0,1)}$). As time progresses, $J_k(t)$ decreases according to (12) since $\lambda _k\ge 0$. The value of $J_k(t)$ decreases to zero over time and when $J_k(t)=0$, a jump associated with ${r}_k$ is triggered (hence the name “jump clock”). Once a jump clock reaches 0 and a jump is triggered, the clock is reset by sampling a new random number, $u_k\sim \text {Unif}(0,1)$. See Fig. 2**C** for a schematic illustration of how the jump clock is updated.

To update the jump clock, we require numerical integration of $\lambda _k(t,\vec {V}(t))$ forward in time. Fortunately, we also have piece-wise polynomial approximations for $\vec {V}_F(t)$ as a result of our numerical treatment of the continuous flows (see Subsection 2.3 ‘Flow events’), combined with piece-wise constant values for $\vec {V}_J(t)$ which only change when jumps occur. We discuss the numerical integration of the jump clock below.

#### Jump clock updates

For a given jump reaction ${r}_k$, a jump clock is initialised at time $t_0$ with $u_k\sim \text {Unif}(0,1)$ giving $J_k = \log (u_k^{-1})$. From time *t* to $t+\Delta t$, with $t>t_0$, Equation (12) tells us the clock “ticks down” from $J_k$ to $J_k - \Delta J_k$, where $\Delta J_k$ is

13 $$\begin{aligned} \Delta J_k = \int _{0}^{\Delta t} \lambda _{k}(t+s,\vec {V}(t+s)) \ \textrm{d}s. \end{aligned}$$

In general this integral is intractable, but we can approximate it as follows: consider the Taylor expansion of $\lambda _{k}(t+s,\vec {V}(t+s))$ about $s = 0$, which gives

14 $$\begin{aligned} \Delta J_k&= \int _{0}^{\Delta t} \lambda _{k}(t,\vec {V}) + \dfrac{\partial \lambda _k}{\partial \vec {V}} \dfrac{\textrm{d}\vec {V}^{\intercal }}{\textrm{d}t} \, s + O(s^2) \ \textrm{d}s = \int _{0}^{\Delta t} \alpha + \beta s + O(s^2) \ \textrm{d}s, \end{aligned}$$

where we write $\alpha = \lambda _{k}(t,\vec {V})$, and $\beta = \dfrac{\partial \lambda _k}{\partial \vec {V}} \dfrac{\textrm{d}\vec {V}^{\intercal }}{\textrm{d}t}$. Since (i) our numerical evaluation of $\vec {V}$ is piece-wise linear across the time interval, by virtue of the fact that we use a forward Euler approximation to solving Equation (7), and (ii) $\Delta t$ is small, $\Delta J_k$ is approximated to the precision of our algorithm by taking

15 $$\begin{aligned} \Delta J_k&\approx \frac{\Delta t}{2} \left( 2\alpha + \beta \Delta t \right) , \end{aligned}$$

noting that since $\vec {V}$ is piece-wise linear across the time interval, the convergence of the integration of the Jump-clock also behaves as $~ \Delta t$. In principle, higher order approximations to the flow events may be utilised, rather than first order forward Euler. However, in these instances, care is required when calculating the gradients for these higher-order schemes, to ensure that no stochastic events occur over the time-step. For this reason, we have limited our current implementation to forward Euler.^3.3^ Importantly, we know that between jumps $\dfrac{\textrm{d}\vec {V}_F}{\textrm{d}t}$ is given by Equation (7) whilst $\dfrac{\textrm{d}\vec {V}_J}{\textrm{d}t}=0$. Thus, $\beta = \dfrac{\partial \lambda _k}{\partial \vec {V}_F} \left( \eta _{FF} \vec {\lambda }_{F}(t,\vec {V}) \right) ^{\intercal }$ (evaluated at *t*). To calculate the updated jump clock, we compute $J_k(t + \Delta t) = J_k(t) - \Delta J_k$ as the provisional value. We then have two distinct cases: (i) $J_k(t) - \Delta J_k > 0$, then no jump occurred during the interval $(t, t+\Delta t)$ and we have the jump clock $J_k(t+\Delta t) := J_k(t) - \Delta J_k$: (ii) $J_k(t) - \Delta J_k < 0$, then a jump occurred (i.e. a ${r}_k$ reaction) during the interval $(t,t+\Delta t)$, that we need to account for in the updated jump clock. In case (ii) where a jump occurs within the interval $(t,t+\Delta t)$, let $t + \Delta \tau$, where $0<\Delta \tau <\Delta t$, denote the time at which this jump occurs. We can find $\Delta \tau$ by interpolation and solving the following for $\Delta \tau$:

16 $$\begin{aligned} 2 \Delta J_k - \Delta \tau (2\alpha + \beta \Delta \tau ) = 0, \end{aligned}$$

where $\Delta J_k$ is the residual of the jump clock from *t* to $t + \Delta \tau$. $\Delta \tau$ is then given by

17 $$\begin{aligned} \Delta \tau = {\left\{ \begin{array}{ll} \frac{\sqrt{\alpha ^2 + 2\beta \Delta J_k} - \alpha }{\beta }, \quad & \beta \ne 0,\\ \frac{\alpha }{\Delta J_k}, \quad & \beta = 0. \end{array}\right. } \end{aligned}$$

If there is a jump, rather than using $\Delta t$ to forward compute the flowing compartments in Equation (8) we instead use $\Delta \tau$ to take part of a flow step and then implement the jump after the flow to get the state at time $t+\Delta \tau$. After this, we reinitialise the jump clock $J_k$ as described above. This procedure is illustrated in Fig. 2**C**.

#### Switching events

The way in which we handle switching is one of the main contributions of this manuscript. Switching events occur when variables transition between $\vec {V}_J$ and $\vec {V}_F$, i.e. between discrete or continuous values. As a consequence, the contents of $\mathcal {R}_J$ and $\mathcal {R}_F$ may change. There are three types of switching events. The first involves a compartment moving from $\vec {V}_J$ to $\vec {V}_F$. This transition is straightforward as a new equation is added to Equation (7), and the state $\vec {V}_F$ is initialised at the switching time, with the new flowing compartment at $\Omega$. Figure 2**D** depicts an example of a type 1 switching event.

The second type involves a flowing compartment moving from $\vec {V}_F$ to $\vec {V}_J$. Here, the flowing compartment reaches the switching threshold, $\Omega$, and becomes discrete and no longer follows the flowing dynamics (of Equation (7)). The time at which this occurs is found by continuation, and computation is resumed. Figure 2**E** depicts an example of a type 2 switching event.

The third type of switching event involves a flowing compartment being changed by a jump event which, unless corrected, would result in the compartment taking a non-integer value below the switching threshold $\Omega$, and hence a transition from $\vec {V}_F$ to $\vec {V}_J$. In this case, a flowing compartment jumps *below* the switching threshold $\Omega$, and becomes discrete.^3.4^ To highlight how the third type of switching event is necessary, we propose considering a simple model where two species, *X* and *Y*, interact to produce a third species, *Z*, with *X* also undergoing a death-dominated birth-death process. Suppose that the switching threshold is set as $\Omega = 1000$, and that $X(0) = 1001$, $Y(0) = 10$ and $Z(0) = 0$. Due to the death reaction of *X*, suppose that within the first (Flow) step, *X* experiences some decay $\varepsilon < 1$. Therefore, $X(t_1) = 1001 - \varepsilon > \Omega = 1000$ is in a flowing (continuous) regime. Now let’s suppose that in the next instance, *X* and *Y* combine to produce one *Z*. At this instance, *X* must experience some integer loss (which we can assume to be 1, but in general may be any positive value). In this case, producing *Z* will result in $X(t_2) < \Omega$ but non-integer. Therefore, to account for type 3 switch events, let $v_i$ be the compartment switching from $\vec {V}_F$ to $\vec {V}_J$ due to a jump. In general, these types of jumps result in $v_i$ being non-integer, i.e. $v_i \notin \{0,1,\ldots ,\Omega \}\cup (\Omega ,\infty )$. To ensure the values of $v_{i}$ stay in $\{0,1,\ldots ,\Omega \}\cup (\Omega ,\infty )$, we add another constraint to the process, initially proposed by Rebuli et al. (2017). The idea is to randomly round the compartment value up or down to an integer in a way that conserves the average behaviour of the process. Let $\hat{v}_i$ be the value of the flowing compartment $v_i$ after jumping down across the threshold $\Omega$ but before being rounded into $\{0,1,\ldots ,\Omega \}\cup (\Omega ,\infty )$. We apply the following rule to round $v_i$ after the switch. We take $v_i = \lceil \hat{v}_i \rceil$ with probability $\hat{v}_i-\lfloor \hat{v}_i \rfloor$, and otherwise we round down by setting $v_i = \lfloor \hat{v}_i \rfloor$. This ensures the expected value of the variable after the switch is $\hat{v}_i$ as described under the flowing paradigm from which this compartment has come and that the variable remains in the domain $\{0,1,\ldots ,\Omega \}\cup (\Omega ,\infty )$. Figure 2**F** depicts an example of a type 3 switching event. Therefore, type 3 switching events will only maintain mass over an ensemble of instances, while type 2 switching events will always maintain mass.^3.4^

Riedler proved a convergence theorem for a general class of algorithms that includes the numerical scheme described above Riedler (2013). This convergence theorem establishes that if $\eta _{FF}\vec {\lambda }_{F}$ and $\sum _{k} \lambda _k$ are time-homogeneous, bounded, Lipschitz continuous and continuously differentiable, and that $\sum _{k} \lambda _k$ is bounded away from zero, then the numerical approximations converges almost surely to the exact samples in the sense that, for the jumps and switches, both the timing of the events and the values of the state converge with order one as $\Delta t\rightarrow 0$. The requirement that the event rate is bounded away from zero is to ensure there can be times at which to consider the convergence, otherwise a purely ODE-based approach to convergence would suffice. The requirement that the system is time-homogeneous can be removed by augmenting the system to include a variable tracking time.^2.3^

## Results

### Effects of interventions to reduce transmission

Simulation-based inference (e.g. the particle filter) relies on being able to efficiently simulate from the generative process. To demonstrate how JSF can be used in this setting, we first present a simulation study of forecasting the elimination of a infectious pathogen.

#### SIRS model with demography

**Fig. 3:**
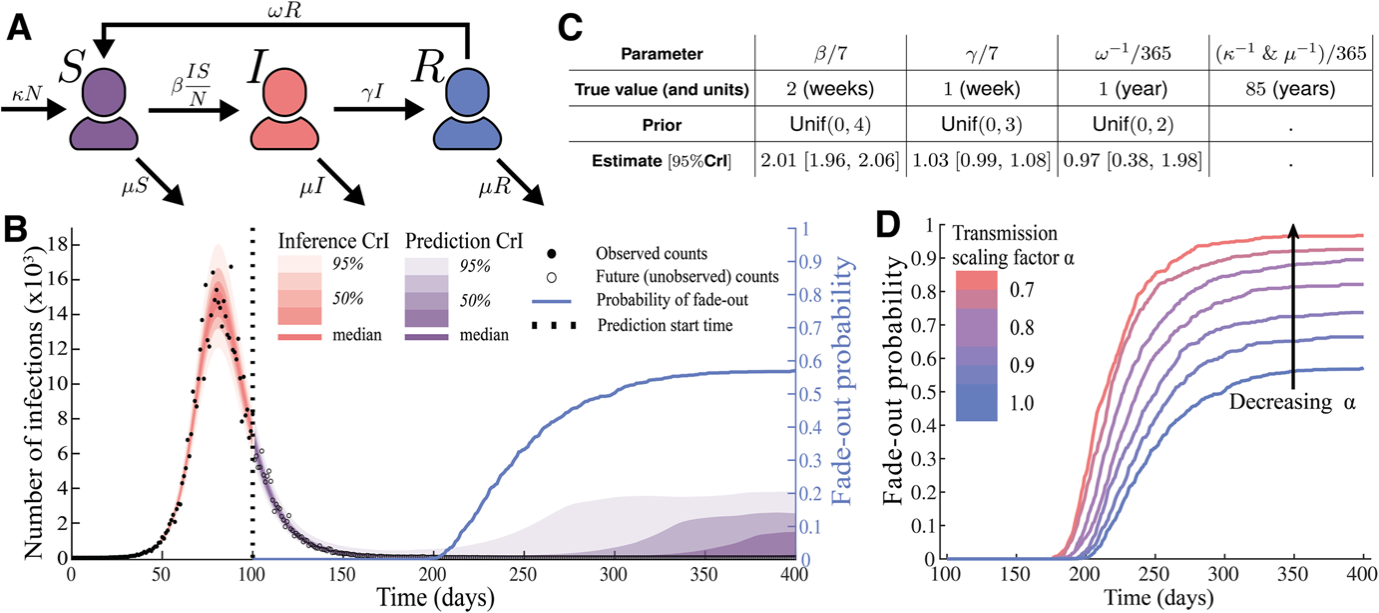
JSF enables us to forecast when a pathogen will be eliminated from a population, i.e. when we can claim that an epidemic has “ended”, and quantify the likely impact of varying levels of intervention. **A** A compartmental model representation of the SIRS with demography. **B** A simulated time series of noisy measurements of prevalence along with inferred prevalence trajectories and forecasts of future prevalence with associated uncertainty bands. The dashed vertical line indicates the date up until which we have data. The solid blue line indicates an estimate of the probability that the pathogen has been eliminated from the population (as opposed to persisting at very low levels). **C** The true parameters used in the simulation along with their posterior estimates and the associated priors used. Each of the marginal posterior distributions peaks tightly about the true value. **D** The probability of pathogen elimination increases with the strength of the intervention against transmission. Near certainty of elimination is achieved with a $30\%$ reduction of transmission

The SIRS model with demography is an extension of the classic susceptible-infectious-removed (SIR) model. In the SIR model *susceptible* individuals may be infected by contact with *infectious* individuals; infected individuals eventually cease to be infectious and transition to the *removed* compartment. The SIRS model with demography extends the SIR model by allowing removed individuals to transition back to being susceptible to infection, by allowing individuals to give birth to new (susceptible) individuals, and by allowing individuals to die (at equal rates). The SIRS model with demography can be written as the following ODE system, with susceptible (S), infectious (I), and recovered (R) individuals:

18 $$\begin{aligned} \begin{aligned} \frac{dS}{dt}&= - \beta \frac{IS}{N} + \omega R + \kappa N - \mu S,\\ \frac{dI}{dt}&= \beta \frac{IS}{N} - \gamma I - \mu I,\\ \frac{dR}{dt}&= \gamma I - \omega R - \mu R, \end{aligned} \end{aligned}$$

where $N(t) = S(t) + I(t) + R(t)$ is the total population size, $\beta$ the infection rate, $\gamma$ the recovery rate, $\omega$ the immunity waning rate, $\kappa$ the birth rate, and $\mu$ the death rate. Figure 3A shows a compartmental diagram of the SIRS model with demography.

Figure 3B shows our simulated (via the Doob-Gillespie algorithm) time series of daily noisy measurements of the prevalence of infection. The true parameters used in the simulation are shown in Fig. 3C; these values are broadly consistent with existing estimates for influenza or SARS-CoV-2. We assume that these measurements are drawn from a negative binomial distribution where the expected value is equal to the true prevalence of infection and there is a constant (known) dispersion parameter, $k=100$.

#### Estimating elimination probabilities

Combining the SIRS model with a particle filter (Moss 2024; Kitagawa 1996) we used the first 100 days of the time series to forecast the remaining 150 days. Figure 3B shows the estimated true prevalence across the first 100 days and forecasts the prevalence for the subsequent 150 days. Included in the forecast is a daily estimate of the probability that the pathogen has been eliminated (in the simulation the pathogen was eliminated on day 250). It is important to note that there is an endemic equilibrium in this model, so it is reasonable for the probability to plateau as it does.

To demonstrate the utility of the posterior distribution (i.e. the capacity of the particle filter to forecast the epidemic after the 100 days of observed data), we considered the impact of a possible intervention. This intervention, introduced on day 100, reduces the force of infection (and hence the effective reproduction number) by a factor of $\alpha$. Figure 3D shows how the probability of eliminating the pathogen increases substantially as we decrease $\alpha$ from 1 (no intervention) to 0.7 (a $30\%$ reduction in transmission). The fade-out probability is calculated as the proportion of sampled (hybrid stochastic-deterministic) trajectories that go extinct (via $I = 0$) of the total number of sampled trajectories.

The full details of the configuration of the particle filter, and the marginal posterior distributions are given in SI Section 2E.

### Computational properties

To further investigate the computational properties of JSF and to compare it with exact (Doob-Gillespie) simulation and (approximate) tau-leaping simulation we used repeated simulations from the SIRS model with demography. We partitioned the simulations into three classes: early stochastic extinction, fade-out after a single epidemic, and sustained transmission (SI Section 2). As seen in Table SI 3, JSF and exact simulation generate very similar proportions of samples in each of these classes. Two aspects of this system of practical importance are the timing and magnitude of peak infections, and the total number of infections. As can be seen in Fig. SI 8 the distribution of peak timing and total number of infections are in good agreement between JSF and exact simulation. For the magnitude of peak infections, there is substantially less variability in the JSF samples than the exact samples, but the proportional difference is small.

With regards to computational efficiency, Fig. SI 11B shows the average time required per simulation using JSF, exact simulation (Doob-Gillespie), tau-leaping, and the efficient Tau-hybrid method Matthew et al. (2023) for the SIRS model for populations of different sizes. For populations of size above about $10^{5}$ (i.e. approximately the size of a small city), JSF is substantially computationally faster than either exact simulation or tau-leaping. Specifically, we observe an order of magnitude reduction in the computational time required to simulate the equivalent physical time horizon, when comparing JSF to the tau-leaping implementation. Moreover, when an appropriate fixed switching threshold is specified, the computational cost remains approximately constant as population size increases, irrespective of compartment occupancy.^2.4^ To simplify the interpretation of the results in Fig. SI 11B, each of the methods were implemented in the same interpreted language so that the observed differences can be attributed to the algorithm rather than the platform on which it is implemented. We have also included a comprehensive simulation case study of the simple birth-death process (see Section SI 2 of the supplementary information), which exhibits JSF^2.5^ computational efficiency for both birth-dominated and death-dominated regimes, with comparable results to the exact stochastic process. We also demonstrate JSF’s accuracy for particularly difficult stochastic simulations which exhibit multiple time scales (see Section SI 3 of the supplementary information), where we see that JSF produces summary statistics and trajectories comparable to the exact stochastic process, out performing current hybrid methods.

### SARS-CoV-2 virus clearance informed by longitudinal data

**Fig. 4:**
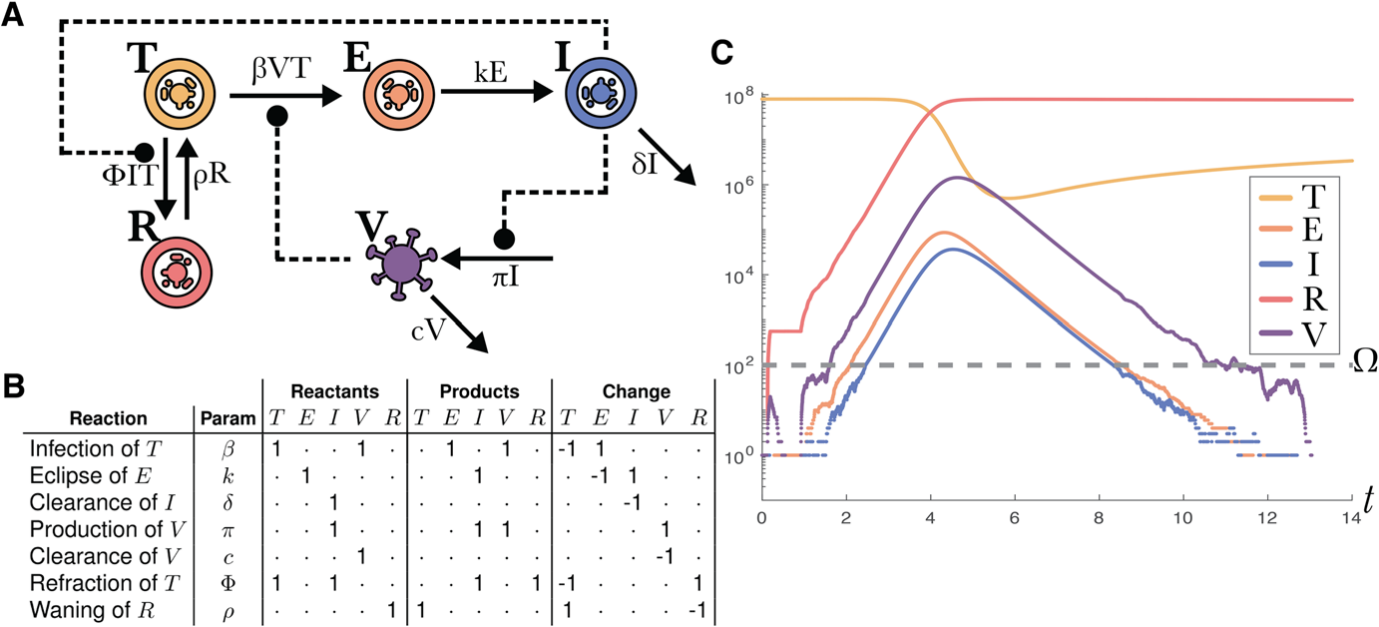
The TEIRV model describes the within-host dynamics of host cells and virus during infection. The model accounts for delays in virus production post cellular infection and the capability of target cells to enter a refractory state as a defence against infection. **A** A compartmental diagram of the TEIRV model showing the interactions between: *Target* cells (*T*), *Refractory* cells (*R*), cells in the *Eclipsed* phase of infection (*E*) and *Infectious* cells (*I*); and the *(Total) Virions* (*V*). Total Virion particles are utilised as to not distinguish between intra- and extra-cellular Virions. Solid arrows represent the exchange of mass (cells or virions) through compartments, while dashed lines represent the influence of a compartment on a reaction’s rate of occurrence. **B** The representation of this model as a reaction network with a stoichiometric matrix. This formalises the dependency on different variables indicated by the dashed arrows in the compartmental diagram. **C** An example trajectory showing the exponential growth and decline in the amount of free virus across the duration of the infection. This process terminates when the compartments representing the virus, and eclipsed^2.6^ and infected cells all reach zero (shortly before time 12). Compartments can hit zero in this hybrid continuous/discrete stochastic model but this does not occur in the solutions of the pure ODE representation

Understanding viral clearance is important when deciding how long treatment must be maintained. This case study demonstrates how JSF enables inference of virus clearance, which has been computationally challenging to date Yan et al. (2016), Farrukee et al. (2018). In particular, we use a mathematical model to study viral clearance using longitudinal data from 6 individuals infected with SARS-CoV-2 Ke et al. (2022). The remainder of this section contains: (i) a description of the TEIRV mathematical model used, (ii) the observation model used to link the cycle number data to viral load, and (iii) key results obtained using JSF to fit the TEIRV mathematical model to the obtained viral load data, using a particle filter.^3.12^

#### TEIRV model of within-host viral dynamics

The TEIRV model is an extension of the classic target-infected-virus (TIV) model Perelson (2002). In the TIV model, *target* cells may be *infected* by the virus; before dying, infected cells produce *virus*; and the virus can degrade or infect the remaining target cells. The TEIRV extends the TIV model through the inclusion of an *eclipsed* compartment, to model the delay between infection of a target cell and the subsequent production of virus, and a *refractory* compartment, to model heightened antiviral defences of target cells, e.g. through the effects of interferons Ke et al. (2022), Blanco-Melo et al. (2020). To simplify the use of this model, a quasi-steady-state approximation for interferon production is employed. This approximation allows us to avoid the need to explicitly model the amount of interferon present.

Figure 4A shows a compartmental diagram of the TEIRV model. Target cells becoming infected by virions at rate $\beta$, which then enter the eclipsed phase. These eclipsed cells then become infectious at rate *k*. Infectious cells are cleared from the population at rate $\delta$, and produce virions at rate $\pi$. Virions are themselves cleared at rate *c*. We note that *V* is the total number of virion particles, and therefore this model does not distinguish between intra- and extra-cellular virions. As a result, following the infection of a target cell (*T*), no virion particles are lost in this model. The infectious cells recruit interferon, which causes^2.6^ the target cells to become refractory (and hence protected against infection) at rate $\Phi$. The refractory cells return to a naive state as target cells at rate $\rho$. These assumptions are represented with the following ODE system:

19 $$\begin{aligned} \begin{aligned} \frac{dT}{dt}&= - \beta V T - \Phi I T + \rho R,\\ \frac{dE}{dt}&= \beta V T - k E,\\ \frac{dI}{dt}&= k E - \delta I,\\ \frac{dV}{dt}&= \pi I - c V,\\ \frac{dR}{dt}&= \Phi I T - \rho R. \end{aligned} \end{aligned}$$

The stoichiometric matrix corresponding to these assumptions is shown in Fig. 4B. Figure 4C shows a trajectory sampled from the TEIRV model when represented with JSF; the classic boom-bust dynamics of the viral populations can be seen in the exponential growth and decline of the *V* compartment. Unlike solutions of the ODE model in (19), we see that by time $t=14$ the populations of eclipsed cells, infected cells, and virus have gone extinct, indicating a definitive end to the infection.

The basic reproduction number, $\mathcal {R}_{0}$, is a fundamental quantity of epidemiological models. For the TEIRV model, $\mathcal {R}_{0}$ can be calculated from the next-generation matrix Diekmann et al. (2010):

20 $$\begin{aligned} \mathcal {R}_0 = \frac{\pi \beta T(0)}{\delta c}. \end{aligned}$$

#### Observation model linking virus to time series

The longitudinal data contains cycle number (CN) values from nasal samples. The CN values are inversely proportional to the number of virions present. In our analysis we used an existing (empirical) model to link the cycle numbers to the (logarithmic) viral genome load Ke et al. (2022): $\log _{10}V = 11.35 - 0.25 \text {CN}$. We assume the observed values are drawn from a normal distribution with mean $\log _{10}V$ and unit standard deviation, and that the values are truncated at the detection limit of $-0.65$.

#### Estimating virus reproduction and clearance

Recall that the particle filter is capable of combining a mechanistic model of the within-host dynamics, such as the TEIRV, and an observation model, such as the viral load measurements, and will return a (Bayesian) posterior sample of both the parameters of the process and the trajectory of virus and cell populations through time. We assume that the infection begins with a single exposed target cell (in a population of $8\times 10^{7}$ target cells) Ke et al. (2022). This gives us an initial condition for the process: $T(0) = 8 \times 10^7$, $E(0) = 1$, $I(0) = 0$, $R(0) = 0$. We leave the initial viral load, *V*(0), as a parameter to be fit to the data.

We selected six patient time series Ke et al. (2022), choosing ones that contained a full 14 data points, for both consistency and simplicity. Two of the model parameters were fixed as in previous analysis Ke et al. (2022): $c=10$, $k=4$. See SI Section 3 for full details of the particle filter and JSF configuration.

**Fig. 5:**
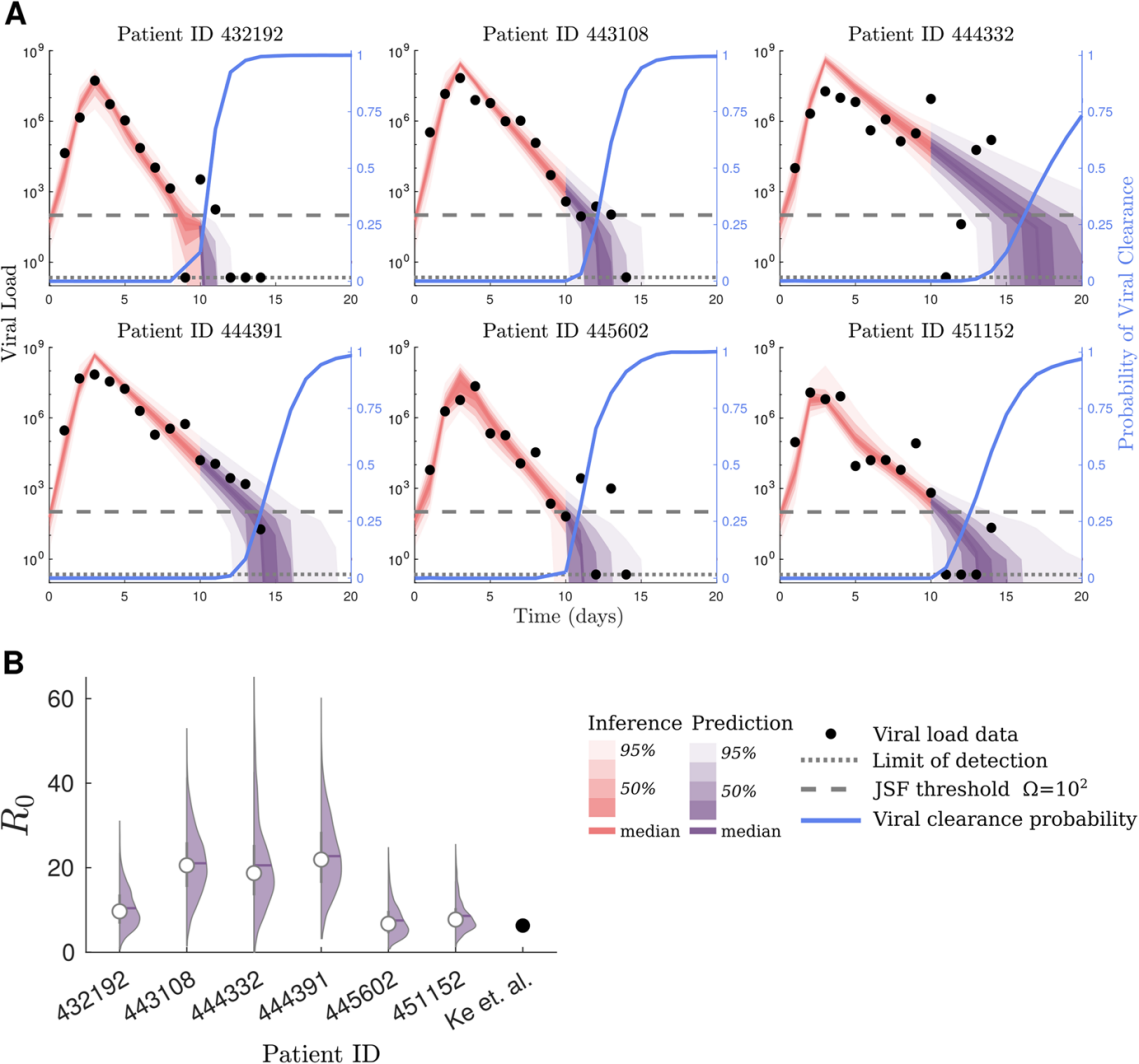
JSF enables the inference of the viral load through time and the probability that the virus has been cleared from longitudinal data of SARS-CoV-2 viral load measurements. **A** Model fits to viral load data based on nasal swabs of six patients from Ke et al. (2022) using a refractory cell within-host model, using our Jump-Switch-Flow method. Nasal viral load data is shown as solid dots, and the limit of detection is shown as a dotted line. We show the switching threshold, $\Omega = 10^2$ by a dashed line. The red and purple bands show mean (0%), 25%, 50%, 75% and 95% credible intervals (from dark to light) for the inference and prediction, respectively. We also estimate the probability of viral clearance at a given point in time, based on the previous data to that point, in blue (light). **B** The posterior distribution of viral $R_0$ for each patient, along with the median (white circle). These patient specific estimates are contrasted with the single point estimate from previous analysis Ke et al. (2022)

Figure 5A shows our model fits to the first 10 days of viral load data for the six selected patients. After day 10, using the estimated parameters, we generate and predict the distribution of subsequent viral load until day 20. The estimated viral peak coincides with the data for each of the patients and the predicted viral trajectories closely match subsequent observations.

We estimate the probability of viral clearance over time (right blue axis of in the panels of Fig. 5A). Viral clearance is defined by the point at which the virion, eclipsed, and infected cell populations all reach extinction. We note that this is a conservative measure if one were only interested in when a patient’s *infectiousness* becomes negligible. However, for proof of concept of inferring such quantities, we have chosen to estimate the probability of complete viral clearance.

There is clear heterogeneity in the amount of time required to clear the virus (Fig. 5A). We estimate that with high certainty patients 423192, 443108 and 445602 clear the virus within 20 days. In contrast, for patients 444332, 444391 and 451152 we infer that they have not cleared the virus within that time frame. Within those who did clear the virus, we observe that different patients require different amounts of time to clear the virus. Specifically, patient 432192 (5A) is inferred to have cleared the virus first. Patients 443108 and 445602 both require an estimated 16 days to obtain viral clearance.

The posterior distributions of the parameters (Fig. SI 12) and priors (Table SI 5), are given in SI Section 5. Because of potential identifiability issues with the rate parameters, we instead compare the estimated reproduction number, $R_0$, to results from previous analysis (Ke et al. 2022). Figure 5B shows the posterior distributions of $R_0$, as well as the point estimates from Ke et al. (2022). Since the previous analysis, (Ke et al. 2022), reports identical estimates for $\pi$, $\beta$ and $\delta$ across these patients, we only include a single point estimate for their work. Our estimate is consistent with previous results (Ke et al. 2022) for patients 432192, 445602 and 451152. However, patients 443108, 444332 and 444391 all have higher $R_0$ estimates in our results. Our $R_0$ estimates are consistent with similar within-host viral infection analyses of other respiratory pathogens (Baccam et al. 2006; Hernandez-Vargas and Velasco-Hernandez 2020; Gubbins 2024).

## Discussion

We have presented a simple hybrid simulation method for compartmental models: *Jump-Switch-Flow* (JSF). This method facilitates efficient simulation of multi-scale models. Through simulation studies and an analysis of longitudinal data from SARS-CoV-2 infections, we demonstrated the desirable computational properties of this method and the types of novel analyses it enables. JSF allows compartments to dynamically change between stochastic and deterministic behaviour. Combining JSF with a simulation-based inference method, e.g. a particle filter, enables inference for multi-scale models, and in situations where absorbing states (such as a population going extinct) are important.

Simulations show JSF produces trajectories largely consistent with gold standard exact simulation techniques, e.g. Doob-Gillespie algorithm (see for example Figs. SI 2–SI 5 and SI 7 – SI 10). We demonstrate how JSF is highly accurate at producing summary statistics comparable to gold standard exact simulation techniques (see Figs. SI 2 – SI 5), with a significant improvement in accuracy for problems with multiple time scales when compared to currently available hybrid methods (see Fig. SI 4 and SI 5). However, JSF is also much faster for realistic population sizes (as can be seen in the comparison of computational speed in Fig. SI 11B). We demonstrate how our approach can be incorporated into a particle filter (Moss 2024) to perform parameter and state estimation, and prediction (see Fig. 3).

A strength of JSF is the capacity to infer when a process has reached an absorbing state, for example, an epidemic fading-out, or a virus being cleared. The ability to infer when an absorbing state has been reached is important for calibrating intervention measures (Parag et al. 2020) and understanding immune response (Yan et al. 2016). ODE-based models rarely have absorbing states that can be reached in finite time, which fundamentally limits their capacity to describe extinction and clearance processes.

We analysed viral load for SARS-CoV-2 infections using the JSF within a particle filter with a TEIRV, refractory cell model (Ke et al. 2022). In the subset of the data considered, we find consistent parameter values between patients (see Section SI 5). In a novel analysis, we estimated the probability of viral clearance through time, finding substantial heterogeneity in the time until viral clearance (see Fig. 5A). This is important as an accurate quantification of when the virus has been cleared is crucial for determining appropriate treatment regimes.

A key advantage of JSF is the ability to combine the stochasticity of discrete small populations with convenient deterministic models for large populations. The point at which a compartment will transition between these descriptions is specified by the threshold parameters $\Omega _{i}$ (one for each compartment). Setting these parameters to low values speeds up computation, while setting them higher captures more of the stochasticity in the process. As demonstrated in the simulation studies, an appropriate value can be determined through some preliminary simulations (see SI 4 for an example of preliminary simulation analysis for the SIRS model example). An important consideration in selecting this parameter is ensuring that the absorbing states can still be reached (e.g. compartment extinction), and that stable and steady states can be perturbed by random fluctuations. Potential considerations when specifying the values $\Omega _i$ may also include preliminary simulations, increasing $\Omega _i$ and observing how the computational complexity scales (see Fig. SI 11). Furthermore, developing theory to better understand when a CTMC is well approximated by an ODE is at the core to determine how the switching threshold should be chosen. We have laid the groundwork for better understanding these processes via numerical simulation-based studies of both the death- and birth-dominant birth-death processes (see Sections SI 2 and SI 3), which demonstrate how the switching threshold $\Omega$ can be fine-tuned to accurately capture the metrics of interest. We also provide an example of how such a numerical simulation-based study may be conducted for an SIRS with demography model (see Section SI 4). In this example, we demonstrate how JSF can be calibrated to exactly (but inefficiently) reproduce the desired metrics of the exact SSA (see Section SI 4A). In Fig. SI 10 we demonstrate how increasing the switching threshold $\Omega$ results in more accurate simulation, and in Fig. SI 11 we demonstrate how decreasing $\Omega$ reduces CPU runtime.^3.1^

The JSF process is a special case of piecewise deterministic Markov processes (PDMP) (Davis 1984). In this work, we have attempted to provide a practical way to instantiate this process for the simulation of compartmental models, in particular by modelling the jumping and switching behaviour appropriately and expressing this framework in a way that is more accessible to computational scientists. While recent work in Riedler (2013), Azaïs et al. (2014), Saporta et al. (2016) has considered methods for simulating PDMPs, it primarily focuses on the general PDMP setting rather than on compartmental modelling.^2.1^ Further understanding of the numerical properties of the Jump-Switch-Flow process and the convergence behaviour of the method for arbitrary choices of flow event solvers will also prove to be an exciting area of further research. However, extending the existing analysis of Riedler (2013) lies outside the scope of this work.^2.1^

Extensions include the incorporation of an intermittent Stochastic Differential Equation approximation between the CTMC and ODE regimes, and extension to spatial processes which exhibit both stochastic and deterministic behaviours.

The modelling framework presented in this paper has the potential to change the way compartmental models are developed and calibrated, moving us towards more accurate and more efficient hybrid methods. These models will help to form the basis of informed decision making, based on realistic and accurate descriptions of the system. This has broad applicability, from ecological models, chemical systems and single cell models, to infectious diseases and within-host models.

## Supplementary Information

Below is the link to the electronic supplementary material.

Supplementary file 1 (pdf 13513 KB)

## Data Availability

The manuscript has associated data in a data repository available at https://github.com/DGermano8/JSFGermano2024. Original data related to SARS-CoV-2 viral load measurements can also be found at Ke et al. (2022).

## Code Availability

All code to reproduce our results is available at https://github.com/DGermano8/JSFGermano2024. A Python package implementing the Jump-Switch-Flow method is available at https://DGermano8.github.io/JSF.
