# Supplementary material for: A hybrid framework for compartmental models enabling simulation-based inference
Source: J Math Biol. 2026 May 27;92(6):93. doi: 10.1007/s00285-026-02409-y (PMC13216187; doi:10.1007/s00285-026-02409-y)
Supplement: Supplementary file 1 — (pdf 13513 KB) [file 285_2026_2409_MOESM1_ESM.pdf]

Supplementary Information: A hybrid framework for compartmental models enabling simulation-based inference

Domenic P.J. Germano [a,b,*], Alexander E. Zarebski [a,c,*], Sophie Hautphenne [a], Robert Moss [d], Jennifer A. Flegg [a], and Mark B. Flegg [e]

[a] *The School of Mathematics and Statistics, The University of Melbourne, Parkville, VIC, Australia*
[b] *The School of Mathematics and Statistics, The University of Sydney, Camperdown, NSW, Australia*
[c] *Pandemic Sciences Institute, University of Oxford, Oxford, United Kingdom*
[d] *Melbourne School of Population and Global Health, The University of Melbourne, Parkville, Vic, Australia*
[e] *School of Mathematics, Monash University, Clayton, VIC, Australia*

SI 1. Jump-Switch-Flow: implementation details

To draw exact samples from the JSF process requires solving the differential equations for the flowing variables $\vec{V}_F$. For nonlinear systems these differential equations are usually intractable, so they are numerically integrated. Here we describe an algorithm for approximately sampling trajectories, assuming we have a numerical solver for the differential equations (when viewed as an initial value problem (IVP)), to approximate the solutions.

SI 1A. Jump-clock

In our code, we implement the JSF algorithm with a slightly different jump clock J_k method than that described in Section 2.3. In particular, we equate $u_k = \text{CDF}(t_k; k)$ but do not take the log of this expression before defining J_k in Equation (12). As a result, in our code we consider the jump clock $\tilde{J}_k$:

$$\tilde{J}_k(t) = u_k - \left(1 - \exp\left\{-\int_{t_0}^{t} \lambda_k(\vec{V}(s))\, \mathrm{d}s\right\}\right), \tag{SI 1}$$

where $u_k \sim \text{Unif}(0, 1)$, and the time of the previous jump event, t_0. Computing the times for the jump events follows a similar process. We compute the jump clock on the regular ODE mesh used to solve $\vec{V}_F$ in Equation (7). From Equation (SI 1) we can write $\tilde{J}_k(t+\Delta t)$ in terms of $\tilde{J}_k(t)$ and an integral of the reaction rate:

$$\tilde{J}_k(t + \Delta t) = \tilde{J}_k(t) + \left(\exp\left\{-\int_{t}^{t+\Delta t} \lambda_k(\vec{V}(s))\, \mathrm{d}s\right\} - 1\right)\left(\tilde{J}_k(t) + 1 - u_k\right). \tag{SI 2}$$

For steps where $\tilde{J}_k(t+\Delta t) > 0$ we can do a single step of this process to get the updated jump clock values. However, if $\tilde{J}_k(t+\Delta t) < 0$, a jump has occurred at some time $t+\Delta\tau \in (t, t + \Delta t)$, and we need to solve $\tilde{J}_k(t+\Delta\tau) = 0$ to find when the jump occurred. Therefore, we require a method to find $\Delta\tau$, where $0 < \Delta\tau < \Delta t$. To do this, we start by letting the right hand side of Equation (SI 2) equal zero and rearrange to get:

$$\int_{t}^{t+\Delta\tau} \lambda_k(\vec{V}(s))\, \mathrm{d}s = \ln\left\{\frac{\tilde{J}_k(t) + 1 - u_k}{1 - u_k}\right\}. \tag{SI 3}$$

If λ_k is a positive constant then:

$$\Delta\tau = \frac{1}{\lambda_k} \ln\left\{\frac{\tilde{J}_k(t) + 1 - u_k}{1 - u_k}\right\}. \tag{SI 4}$$

*These authors contributed equally to this work

If λ_k is not constant, we approximate λ_k, which we call $\widehat{\lambda}_k$, using the integration of $\vec{V}(t)$, as per Equation (7), since we know $\vec{V}(t)$ and $\vec{V}(t+\Delta t)$. Therefore, we also know λ_k at t and $t+\Delta t$. We then write $\widehat{\lambda}_k$, at $t+\Delta\tau$ between times times t and $t+\Delta t$ via linear interpolation:

$$\widehat{\lambda}_k(t+\Delta\tau) \approx \left(1-\frac{\Delta\tau}{\Delta t}\right)\lambda_k(\vec{V}(t)) + \frac{\Delta\tau}{\Delta t}\lambda_k(\vec{V}(t+\Delta t)). \tag{SI 5}$$

We can now substitute this approximation into Equation (SI 3), solve it, and rearrange for $\Delta\tau$. This gives a quadratic equation in $\Delta\tau$ with the following solution:

$$\Delta\tau = \frac{\sqrt{\left(\Delta t\widehat{\lambda}_k(t)\right)^2 + 2\alpha\Delta t\Delta\lambda_k} - \Delta t\widehat{\lambda}_k(t)}{\Delta\lambda_k}, \tag{SI 6}$$

where we have introduced the shorthand $\Delta\lambda_k = \lambda_k(\vec{V}(t+\Delta t)) - \lambda_k(\vec{V}(t))$.

SI 1B. Pseudocode details

As with a typical IVP, we require the following data:

- the initial condition of the system, $\vec{V}(0)$;
- the final time $T_{\max} > 0$;
- the stoichiometric reactant matrix, η^-, and product matrix, η^+, from which we derive the exchange matrix, $\eta = \eta^+ - \eta^-$;
- a set of the associated propensities of the reactions, $\mathcal{R}$;
- a time step size for the numerical integrator, Δt;
- a vector of switching thresholds, $\mathbf{\Omega}$;

To sample the Jump-Switch-Flow process, we have identified both an *exact* sampler, that samples the process exactly, and an *operator-splitting* sampler, that uses some simplifying approximations that makes sampling substantially faster.

Exact Jump-Switch-Flow sampler

Here, we describe an *exact* algorithm for sampling trajectories from Jump-Switch-Flow. The algorithm to sample *exactly* from Jump-Switch-Flow is given in Algorithm SI 1. First, initialise the jump clocks (line 3). On each iteration through the ODE loop, we first partition the reactions based on inclusion in $\mathcal{S}$ (line 6). We use the IVP solver to compute $\Delta\vec{V}_F(t)$ based on the flowing reactions (line 7). For each step of the ODE loop, we update the jump clocks (line 9). On lines 10–12, we determine if a jump event has occurred based on the sign of the the Jump-clock. If a jump event has occurred, compute the corresponding event time. On line 13 we then rewind the state (and jump clocks) to when the jump occurred. Resample the appropriate jump clock (line 14), and update the state to account for the jump (line 15). Repeat until the time has reached the maximum desired time.

Operator Splitting Jump-Switch-Flow sampler

Here, we describe the algorithm for the *operator-splitting* algorithm, for sampling trajectories from Jump-Switch-Flow, which utilises some simplifying approximations to improve sampling performance.

The Operator Splitting Jump-Switch-Flow sampler is described in Algorithm SI 2. Unlike the Exact JSF sampler, when a jump event occurs, we continue to integrate the system with the same flow event, $\Delta\vec{V}_F(t)$, instead of resampling it as before. This, in turn, ensures that the ODE is not continually being resampled. While this adds complexity at the implementation stage, significant computational time saving is achieved.

We first initialise the Jump-Switch-Flow process by initialising all the jump clocks (line 3). On each iteration through the ODE loop, we first partition the reactions based on inclusion in $\mathcal{S}$ (line 6). We use the IVP solver to compute $\Delta\vec{V}_F(t)$ based on the flowing reactions (line 7). We now iterate through this current time-step, Δt, checking if any jump events occur (lines 10 – 24), named the *jump loop*. First, we track how much "relative time" (δt) passes through this jump loop (line 9). We then update the jump clocks to

Algorithm SI 1 An algorithmic description of the Exact Jump-Switch-Flow sampler.

Require: $\vec{V}(0)$, $\Delta t > 0$ and $T_{\max} > 0$
1: Initialise model state
2: $t \leftarrow 0$
3: Initialise jump clocks $u_i \sim \mathrm{Unif}(0,1)$ for $i = 1, \ldots, N$
4: ($\triangleright$ Start ODE loop)
5: **while** $t < T_{\max}$ **do**
6: Compute jumping reactions $\mathcal{S}(t)$
7: Compute flow event, $\Delta \vec{V}_F(t)$
8: ($\triangleright$ Perform jump loop for ODE mesh step)
9: Update jump clocks, $\tilde{J}_k$, to $t + \Delta t$ ($\triangleright$ As per Equation (15))
10: **if** any $\tilde{J}_k \leq 0$ ($\triangleright$ Reaction Occurred) **then**
11: Identify first fired Reaction, j
12: Compute time of jump event, $\Delta\tau$ ($\triangleright$ As per Equation (17))
13: Reverse jump clocks to time $t + \Delta\tau$
14: Resample $u_j \sim \mathrm{Unif}(0,1)$
15: Update both: $\vec{V}_J(t+\Delta\tau) = \vec{V}_J(t) + \eta_j$ **and** $\vec{V}_F(t+\Delta\tau) = \vec{V}_F(t) + \Delta\tau\, \Delta\vec{V}_F(t) + \eta_j$
16: $t \leftarrow t + \Delta\tau$
17: **else if** $\tilde{J}_k > 0, \forall k$ **then**
18: Update: $\vec{V}_F(t+\Delta t) = \vec{V}_F(t) + \Delta t\, \Delta\vec{V}_F(t)$
19: $t \leftarrow t + \Delta t$
20: **end if**
21: **end while**

the end of the jump loop, accounting for any relative time that has occurred (line 11). As with the Exact JSF sampler, on lines 12 – 14, we determine if a jump event has occurred using the Jump-clock. This gives us which Reaction has occurred, and at what time, $t + \Delta\tau$. On line 15 we then reverse the system to the time the jump event occurs, noting that we must reverse the excess between when the jump event occurred and the remainder of the time-step. We then resample the associated jump clock that just fired (line 16), and update the whole state (i.e. both flowing and jumping variables) to the current time (line 17). We update the current time to time $t + \Delta\tau$ (line 18) and track the relative time in the jump loop (line 19). We then continue through the jump look. If no jump events have occurred, we leave the loop (line 21). We then simply update the whole state according to the flow event calculated, accounting for any time spent inside the jump loop (line 24).

Algorithm SI 2 An algorithmic description of the Operator Splitting Jump-Switch-Flow sampler .

Require: $\vec{V}(0)$, $\Delta t > 0$ and $T_{\max} > 0$
1: Initialise model state
2: $t \leftarrow 0$
3: Initialise jump clocks $u_i \sim \mathrm{Unif}(0,1)$ for $i = 1, \ldots, N$
4: ($\triangleright$ Start ODE loop)
5: **while** $t < T_{\max}$ **do**
6: Compute jumping reactions $\mathcal{S}(t)$
7: Compute flow event, $\Delta \vec{V}_F(t)$
8: ($\triangleright$ Perform jump loop for ODE mesh step)
9: Set $\delta t \leftarrow 0$
10: **while** $\Delta t > \delta t$ **do**
11: Update jump clocks, $\tilde{J}_k$, to $t + (\Delta t - \delta t)$
12: **if** any $\tilde{J}_k \leq 0$ ($\triangleright$ Reaction Occurred) **then**
13: Identify first fired Reaction, j
14: Compute time of jump event, $t + \Delta\tau$
15: Reverse jump clocks to time $t + \Delta\tau$
16: Resample $u_j \sim \mathrm{Unif}(0,1)$
17: Update both: $\vec{V}_J(t+\Delta\tau) = \vec{V}_J(t) + \eta_j$ **and** $\vec{V}_F(t+\Delta\tau) = \vec{V}_F(t) + \Delta\tau\, \Delta\vec{V}_F(t) + \eta_j$
18: $t \leftarrow t + \Delta\tau$
19: $\delta t \leftarrow \delta t + \Delta\tau$
20: **else if** $\tilde{J}_k > 0, \forall k$ **then**
21: Leave while loop
22: **end if**
23: **end while**
24: Update: $\vec{V}_F(t + (\Delta t - \delta t)) = \vec{V}_F(t) + (\Delta t - \delta t)\, \Delta\vec{V}_F(t)$
25: $t \leftarrow t + (\Delta t - \delta t)$
26: **end while**

SI 1C. JSF Error analysis

To investigate the *global truncation error* of JSF over some finite time horizon, we consider the size of a population through time $X(t)$, governed by a simple birth-death process, undergoing growth-dominated dynamics. In this case, we know the exact deterministic values of $X(t)$, and so can compare the know expected value at some finite time, $t = t_{\max} = 1$. We specify a growth rate of $\lambda = 1$, and a death rate of $\mu = 0.5$, and an initial population size of $x(0) = 10$. Figure SI 1 shows the global truncation error to be $\mathcal{O}(\Delta t)$, as we implement the flow events via a Forward Euler method.

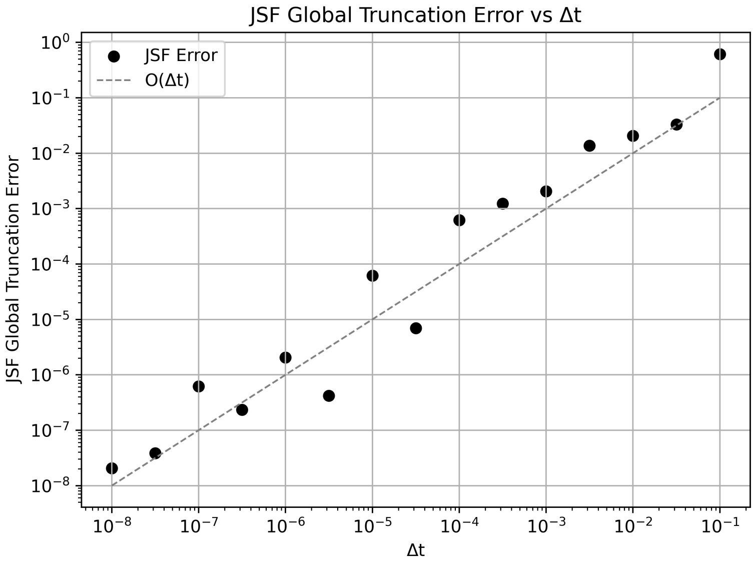

Figure SI 1: Global truncation error of the JSF method over a finite time horizon, computed by comparing the forward Euler numerical solution to the exact solution of the birth-death process.

SI 2. Simulation Study: Birth-death processes

Fundamentally, one can conceptualise any compartmental model as a collection of interacting birth-death processes. For this reason, we will consider two very simple such processes: 1) a birth-dominated birth-death process, and 2) a death-dominated birth-death process. In this section, we will perform a simulation study to highlight the advantages of our Jump-Switch-Flow method, and compare the results to the gold standard, Doob-Gillespie Exact method, and the highly computationally efficient Tau-hybrid method, which is provided by GillesPy2, written in both C++ and Python.

The exact model we will utilise is a single species, X, experiencing birth with rate α, which results in $X \to 2X$, and death with rate β, which results in $X \to \varnothing$.

A birth-dominated birth-death process

For the birth-dominated process, we require $\alpha > \beta$. Therefore, we specify $\alpha = 1$, and $\beta = 0.5$. We also choose the initial species population $X(0) = 1$, and a simulation time of $t = 20$. The quantities of interest to test the accuracy and efficiency of the methods are (i) the CPU time for a particular simulation instance, (ii) the simulation time required to reach a particular value, $X = 200$, and (iii) the accuracy of the method to experience initial population extinction. Figure SI 2 shows results for each of the three quantities of interest for the Tau-hybrid method (red), the Doob-Gillespie method (yellow) and three different Jump-Switch-Flow configurations (purple) with varying switching thresholds of $\Omega = 10^1$, $\Omega = 10^2$, and $\Omega = 10^3$, respectively. Here, 1000 unique trajectories are considered for each method.

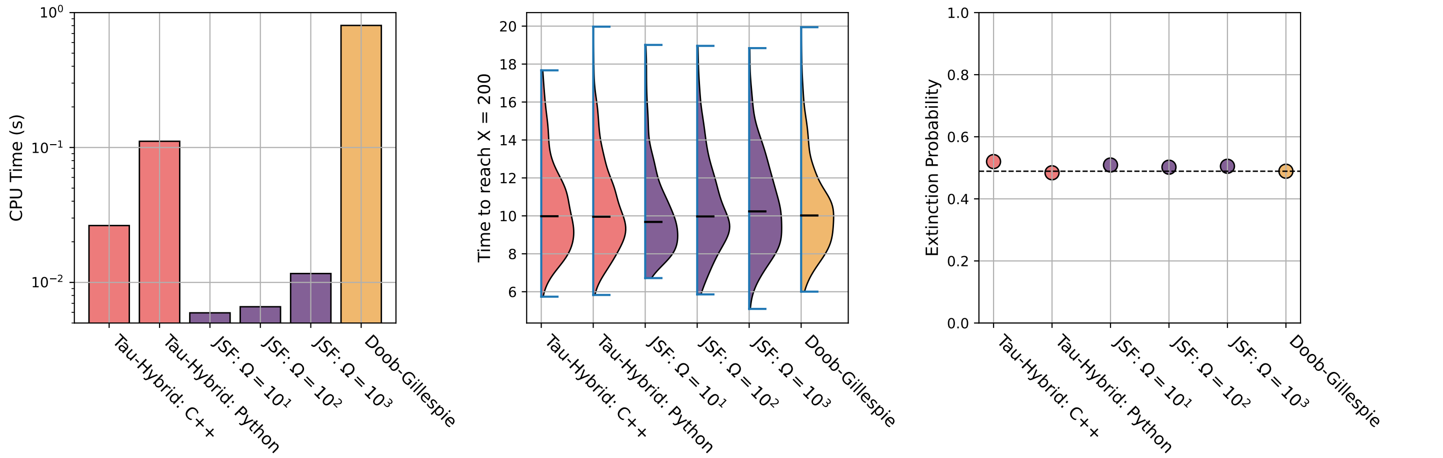

Figure SI 2: Summary statistics for the birth-dominated birth-death process for the Tau-hybrid (red), JSF for various switching thresholds, Ω, (purple) and the Doob-Gillespie (yellow) methods. Left: CPU time required. We observe that as Ω increases, the computational time of JSF increases, but JSF remains the computationally least expensive. Middle: Distributions of time to reach $X = 200$. We observe that JSF results in comparable distributions to the exact samples (generated by Doob-Gillespie). Right: Extinction probability. We observe that all point estimates are comparable to that of the exact method.

Considering the CPU time, we can observe that, as we increase the switching threshold, JSF requires more CPU time, since more of the simulation is in a discrete, jumping regime. We also observe that the CPU time is, at worst, around two orders of magnitude less for JSF than that of the Doob-Gillespie method. In comparison, we observe that the C++ implementation of Tau-hybrid method requires at best three times the computational time to simulate the equivalent process as JSF, while the Python implementation is at least one order of magnitude slower, indicating that JSF is much more efficient. To assess the accuracy of each method, we now compare the (simulation) time for the species to reach 200. Here, both Tau-hybrid and JSF with switching thresholds of $\Omega = 10^2$, and $\Omega = 10^3$ result in distributions and median value comparable to that of Doob-Gillespie. The only exception is that of JSF with a switching threshold of $\Omega = 10^1$. Lastly, we observe that all methods result in an initial extinction probability comparable to that of Doob-Gillespie.

These results suggest that JSF is capable of recovering the key quantities of the exact stochastic method, while being computationally cheaper than current hybrid methods and the exact method, for the birth-dominated birth-death process.

A death-dominated birth-death process

For the birth-dominated process, we require $\alpha < \beta$. Therefore, we specify $\alpha = 0.5$, and $\beta = 1$. We choose the initial species population $X(0) = 100$, and a simulation time of $t = 30$. As before, the quantities of interest to test the accuracy and efficiency of the methods are (i) the CPU time for a particular simulation instance, (ii) the simulation time required to reach a particular value, $X = 50$, and (iii) the simulation time require for the species to go extinct. Figure SI 3 shows results for each of the three quantities of interest for the Tau-hybrid method (red), Doo-Gillespie (yellow) and three different Jump-Switch-Flow configurations (purple) with varying switching thresholds of $\Omega = 10^1$, $\Omega = 10^2$, and $\Omega = 10^3$, respectively. Again, 1000 unique trajectories are considered for each method. Unlike in the previous example, here we observe that all JSF configurations require more CPU time to complete the equivalent computation. However, we still observe that all JSF methods require less CPU time when compared to the C++ implementation of the Tau-hybrid method, this time consuming at least 1.5 times less computation time, and still at least one order of magnitude quicker when compared to the Python implementation. We also note that JSF requires at most half the computational time of that of the exact Doob-Gillespie method. We now consider the time for the species to reach a population of size $X = 50$. In this example, we see that JSF with $\Omega = 10^1$ does not capture the distribution of the exact method, but only reports a single value. This is due to trajectories from this example all being deterministic at this value. If we consider JSF with $\Omega = 10^2$, we see that while the median is comparable to the exact method, the distribution is more concentrated. This is because the method only becomes stochastic at $X = 100$, resulting reduced variance in the trajectory prior to this value. As we increase the switching threshold to $\Omega = 10^3$, the distribution of the time to reach $X = 50$ becomes comparable to that of the exact method. This provides a clear guide for how the switching threshold may be chosen, given some prior knowledge of the system. Lastly, we observe that the extinction time for the JSF with $\Omega = 10^2$ and $\Omega = 10^3$ results in a comparable distribution to that of the Doob-Gillespie method. Again, we also observe that JSF with $\Omega = 10^1$ results in more concentrated distribution to that of the exact method.

These results also suggest that JSF is capable of recovering the key quantities of the exact stochastic method, while being computationally cheaper than current hybrid methods, for the death-dominated birth-death process. Through this example, we also show how

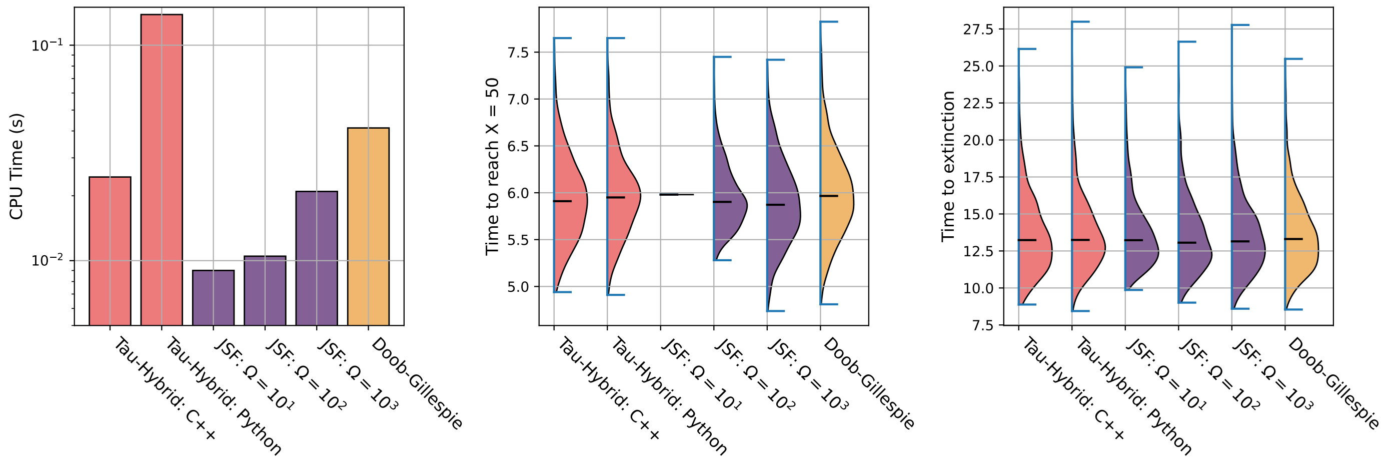

Figure SI 3: Summary statistics for the death-dominated birth-death process for the Tau-hybrid (red), JSF for various switching thresholds, Ω, (purple), and the Doob-Gillespie (yellow) methods. Left: CPU time required. We again observe that, as Ω increases, the computational cost of JSF increases, but it still remains the least expensive computationally. Middle: Distributions of the time to reach $X = 50$. We observe that a switching threshold of $\Omega = 10^3$ is required for JSF to result in comparable distributions to the exact process, Right: Distribution in the time to extinction. We observe that for $\Omega > 10^1$, JSF results in comparable distributions to that of the exact method.

JSF may exhibit sensitivity in the value of the switching threshold chosen, and how to circumvent these sensitivities.

SI 3. Simulation study: Multiple time-scales in autocatalysis

To emphasise the utility and accuracy of our Jump-Switch-Flow method, we will consider a simple autocatalysis example, with multiple time-scales. Consider two species, X and Y, which react together via the following reactions: $X + Y \to 2X + Y$ with rate α, $X \to 2X$ with rate β, $X \to \varnothing$ with rate γ, and $Y \to \varnothing$ with rate δ. To demonstrate the ability of JSF to simulate the trajectories with multiple time-scales, but importantly, simulate them *accurately*, we choose the following rate parameters:

1. a moderate growth rate of X: $\beta = 10$,

2. a large death rate of Y, to ensure we cannot assume a constant Y: $\delta = 100$,

3. a large death rate of X, meaning X quickly vanishes: $\gamma = 50$,

4. and lastly a small autocatalysis rate, resulting in X varying drastically in scale: $\alpha = 1$.

Despite being a relatively simple model with an analytic deterministic description, simulating stochastic trajectories requires careful consideration of the partitioning between the stochastic reactions to ensure an accurate representation, as there are reactions that are occurring a multiple different timescales. For example, autocatalysis occurs initially relatively frequently, due to the large number of species Y, despite the small rate α. However, following the rapid decline of Y, X will experience moderate decay. Therefore, we expect to observe two different timescales in the species X: a rapid increase, followed by gradual decline.

We assign the initial species sizes $X(0) = 10$, and $Y(0) = 10^2$, and sample 1000 trajectories using the exact Doob-Gillespie method, the GillesPy2's C++ hybrid method (Tau-hybrid), and JSF with a switching threshold of $\Omega = 10^2$. Figure SI 4 shows the 1000 trajectories for Tau-hybrid, JSF (with $\Omega = 10^2$), and Doob-Gillespie. We also present the analytic ODE solutions (dashed black) and the median value at a point (solid black).

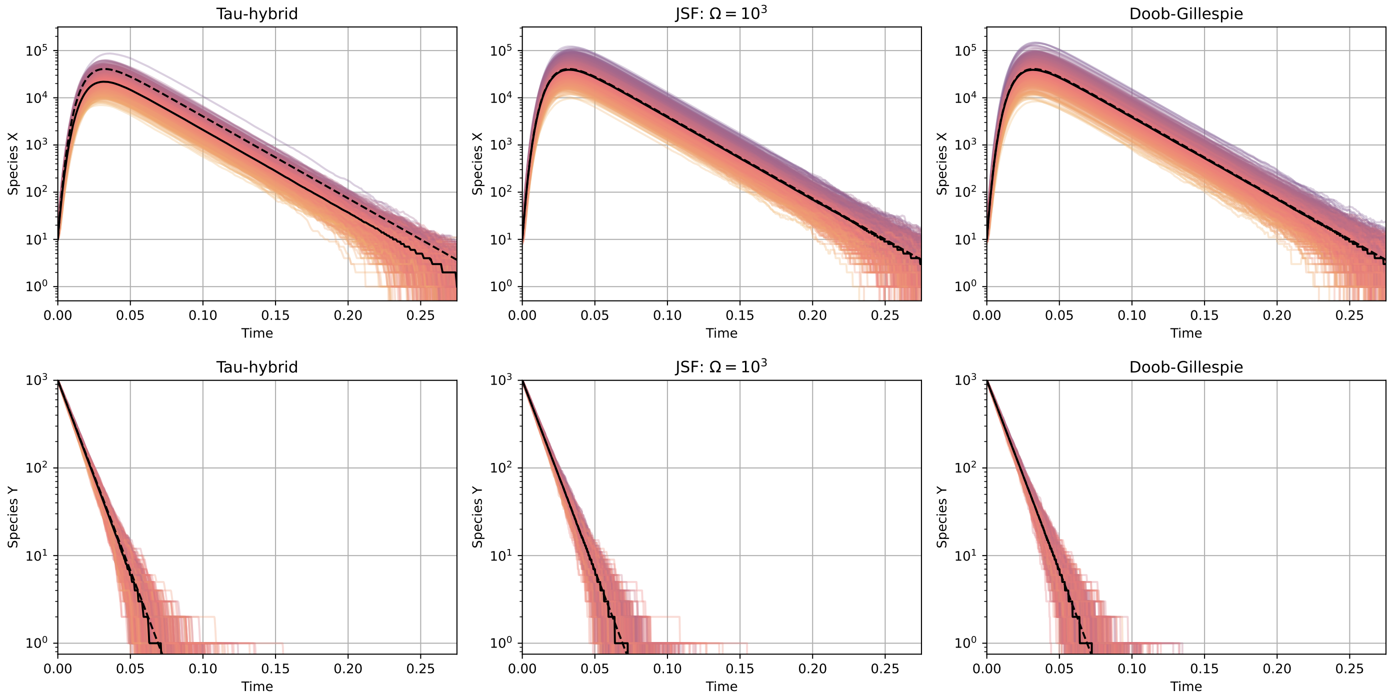

Figure SI 4: 1000 trajectories for the multiscale autocatalyst example for the Tau-hybrid method (left), the JSF method (middle) and the exact Doob-Gillespie method (right). Top shows species X, and bottom shows species Y. We also show the median species value (solid black line) and the analytic ODE solution (dashed black line). We see that the median trajectory of Tau-hybrid is less than the analytic solution, while both Doob-Gillespie and JSF match the analytic solution.

Here, we can see that all Y species solutions match the analytic solution, since species Y undergoes constant decay only. However, we clearly see that, while the median trajectories of JSF and Doob-Gillespie match that of the analytic description, the Tau-hybrid method median is less than the analytic solution. We can further quantify these discrepancies by looking at the distributions of the time for species X to reach a particular value, the distributions of the maximum X value, and also the relative point error between the median of the particular approach, and the analytic solution. We present these quantities in Figure SI 5.

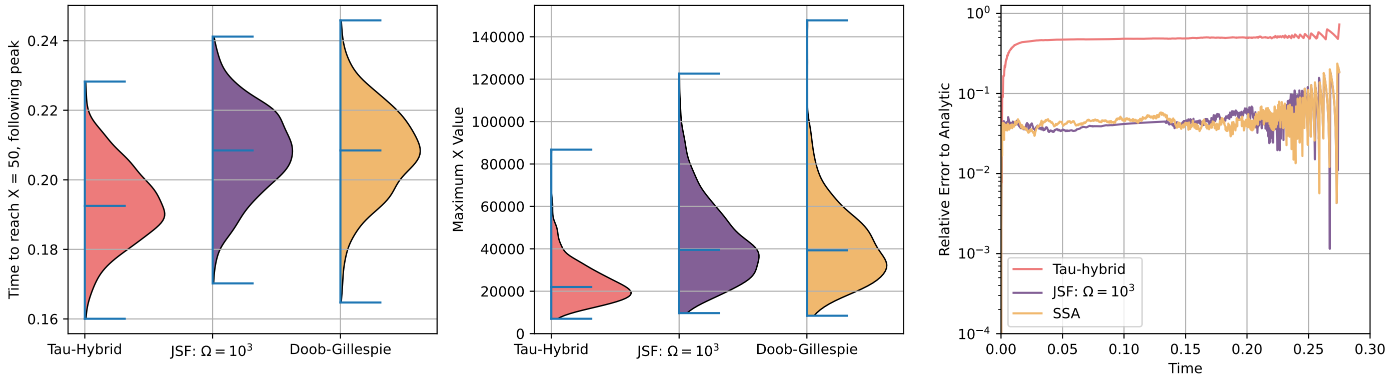

Figure SI 5: Summary statistics for the multiscale autocatalyst example for the Tau-hybrid method (red), JSF method with $\Omega = 10^3$ (purple), and Doob-Gillespie method (yellow). Left: time to reach $X = 50$ following initial peak. We see that the distributions of Doob-Gillespie and JSF are comparable, while tau-hybrid is skewed downward with a different median value. Middle: Maximum X value. Again, we observe JSF and Doob-Gillespie resulting in matching distributions, while Tau-hybrid is skewed downwards. Right: relative error to analytic solution. JSF and Doob-Gillespie display similarly small error, an order of magnitude less than Tau-hybrid.

Figure SI 5 shows the distributions of the time to reach $X = 50$ following the peak, where we see that JSF and Doob-Gillespie produce comparable results. However we also observe that the Tau-hybrid method under-performs, and results in earlier hitting times. We observe a similar trend in the maximum X value, with Tau-hybrid producing a skewed downwards distribution, while JSF and

Doob-Gillespie producing comparable distributions. Lastly, the relative point error of JSF and Doob-Gillespie are comparable, with Tau-hybrid being an order of magnitude greater.

From the results presented here, we see that our Jump-Switch-Flow method is capable of producing highly accurate hybrid trajectories, even with highly multiscale systems, as the one presented here. We also observe the summary statistics produced by JSF being comparable to those of the exact Doob-Gillespie method, and outperforming the highly efficient and sophisticated Tau-hybrid method.

SI 4. Simulation study: SIRS with demography

We first present how the SIRS model with demography can be expressed with the Jump-Switch-Flow method. For the Jump-Switch-Flow method to be a useful method, it must reproduce behaviour that is representative of the exact process (i.e. the CTMC). That is, given some summary statistic, distributions produced via sampling the JSF process should be comparable to those obtained via sampling the CTMC. Moreover, we require that it does so with acceptable computational efficiency. We show, through simulation experiments, how the Jump-Switch-Flow method compares to the Doob-Gillespie method [1] for accuracy, and how our approach can exhibit sensitivity with respect to model inputs. We present how our Jump-Switch-Flow method compares to both the Doob-Gillespie and Tau-Leaping methods.

Figure SI 6a shows the main reactions of the SIRS model with demography: individuals fall into one of three compartments: susceptible (S), infectious (I) and recovered (R). Here, arrows represent how individuals move and progress through compartments. Specifically, individuals are born into the susceptible compartment. Births occur from individuals in the S, I and R compartments, and therefore depend explicitly on the populations (discrete or continuous) in each of these compartments. Individuals can die within each of the compartments. Finally, individuals may progress through compartments via susceptible individuals experiencing infection, infected individuals experiencing recovery, and recovered individuals experiencing waning immunity.

In order to represent the SIRS model with demography as a Jump-Switch-Flow process, we need to associate rates, reactants and products to each of the arrows (see Figure SI 6b). Note that the births arrow is split into three arrows depending upon the compartment of the parent. Moreover, one of the key assumptions of any SIR-type model is that the population is homogeneously mixed, which enables us to describe how individuals interact with one another. This enables us to say that susceptible individuals interact with infectious individuals with a rate inversely proportional to the whole population. However, there are likewise infectious individuals interacting with other infectious individuals, and also with recovered individuals. However, these last two interactions (as shown in blue in Figure SI 6b) do not result in any exchange of individuals between compartments.

Figure SI 6c shows the SIRS model with demography as a Jump-Switch-Flow process. In this example figure, the S and R compartments are continuous (flowing), while the I compartment is discrete (jumping). To decide which of the arrows/reactions are jumping, i.e. in $\mathcal{S}$, and which are flowing, i.e. in $\mathcal{S}'$, we consider the reactants and products of the arrows: if an arrow has any discrete reactant or product, then that arrow is also jumping, otherwise it is flowing. Table SI 1 shows the reactants and products of the SIRS model with demography.

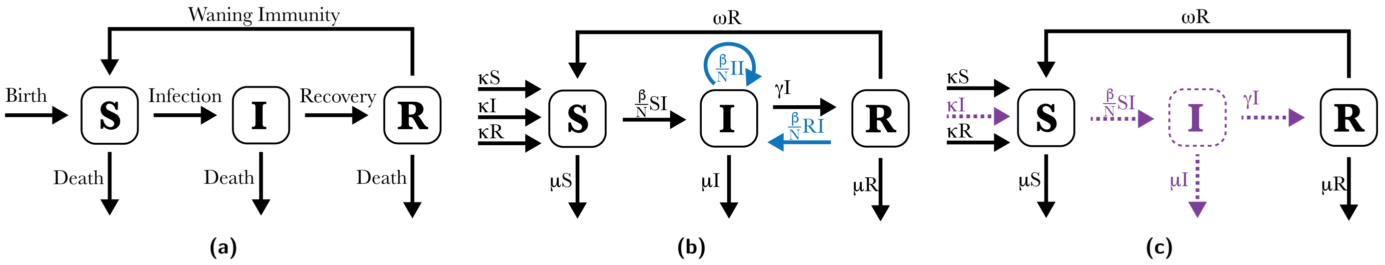

Figure SI 6: (a) The labelled compartmental SIRS model with demography. (b) The SIRS model with demography where the arrows describe the interactions between the reactants and products. Here, blue arrows do not result in any flow through the system, and can be ignored. (c) The model as a Jump-Switch-Flow system with continuous (flowing) S and R (black), and discrete (jumping) I (purple). Since I is jumping, the reactions involving I are modelled as discrete, jumping reactions.

For the remainder of this section, we suppose parameter values for the SIRS model with demography that would be typical for a newly introduced, yearly seasonal communicable disease, as presented in Table SI 2. With these parameter values, on average, we expect an infected individual to infect 2 other people per week (during the initial outbreak), and be infectious for 1 week, with immunity to the disease lasting for 1 year. For the population turnover, we assume individuals live for, on average, 85 years. Initially there are two infectious individuals (i.e. $I(0) = 2$), no recovered individuals (i.e. $R(0) = 0$), and the remainder of the population is susceptible (i.e. $S(0) = N(0) - I(0)$).

		Reactants, η^-			Products, η^+			η		
**Reaction**	**Rate**	S	I	R	S	I	R	S	I	R
Birth by S	κ	1	·	·	2	·	·	1	·	·
*Birth by I	κ	·	1	·	1	1	·	1	·	·
Birth by R	κ	·	·	1	1	·	1	1	·	·
*Infection of S	β_S/N	1	1	·	·	2	·	-1	1	·
*Infection of I	β_I/N	·	2	·	·	2	·	·	·	·
*Infection of R	β_R/N	·	1	1	·	1	1	·	·	·
*Recovery of I	γ	·	1	·	·	·	1	·	-1	1
Waning of R	ω	·	·	1	1	·	·	1	·	-1
Death of S	μ	1	·	·	·	·	·	-1	·	·
*Death of I	μ	·	1	·	·	·	·	·	-1	·
Death of R	μ	·	·	1	·	·	·	·	·	-1

Table SI 1: The stoichiometric matrices for the SIRS model with demography. The (*) indicates reactions that are jumping when I is discrete and S and R are continuous. The rows with blue coloured entries do not alter the exchange through the system, and so need not be represented.

Parameter	β	γ	ω	κ	μ
Rate description	Infection	Recovery	Immunity Waning	Birth	Death
Value	2/7	1/7	1/365	$1/(85 \times 365)$	$1/(85 \times 365)$

Table SI 2: Parameter values used for SIRS model with demography.

As well as being computationally efficient, our Jump-Switch-Flow method is capable of capturing the inherent stochastic nature of the sampled process. For the SIRS model with demography, this is realised by three key different scenarios:

1. *Extinction*: all of the individuals recover before the disease manages to spread significantly throughout the population (see Figure SI 7a and SI 7d);

2. *Fade-out*: the disease spreads throughout the population, however it fades-out in the trough proceeding the first wave (see Figure SI 7b and SI 7e);

3. *Endemic*: the disease persists indefinitely within the population (see Figure SI 7c and SI 7f).

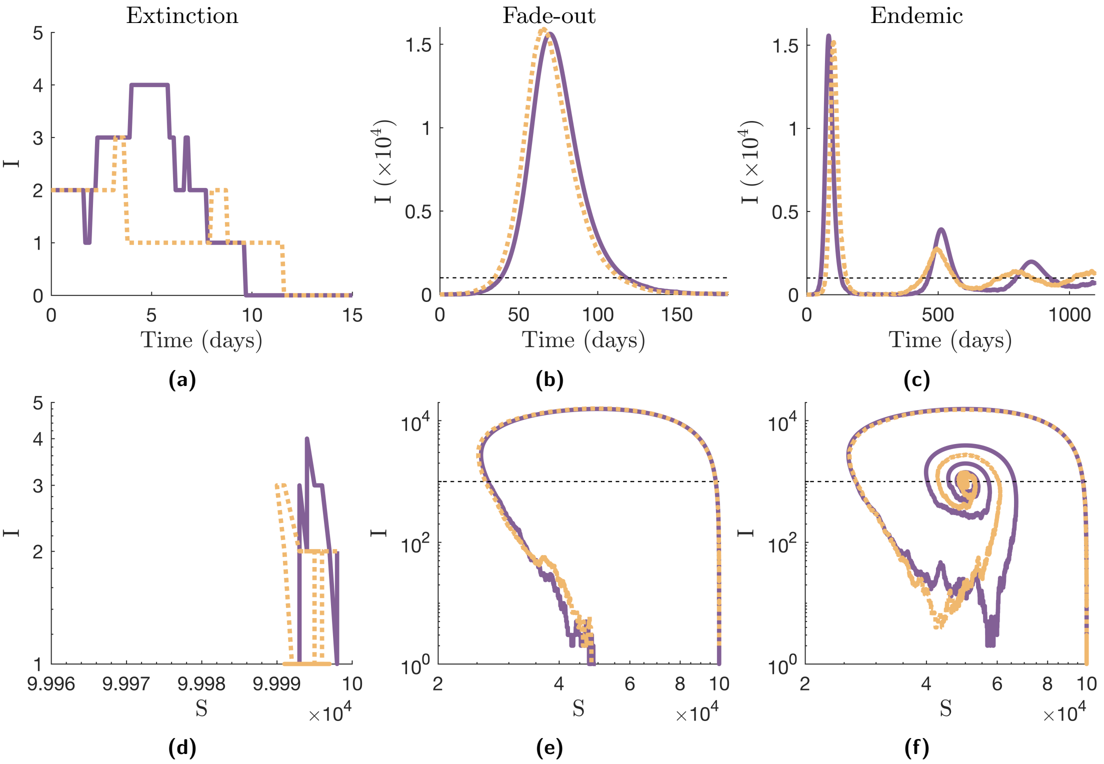

Figure SI 7: Possible simulated trajectories of SIRS model with demography for an initial population size $N(0) = 10^5$, from Doob-Gillespie (yellow) and Jump-Switch-Flow (purple, $\Omega = 10^3$) methods: (a) two examples of extinction trajectories of the infectious compartment, with associated $S - I$ phase plane in (d); (b) two examples of fade-out trajectories of the infectious compartment, with associated $S - I$ phase plane in (e); (c) two examples of endemic trajectories of the infectious compartment, with associated $S - I$ phase plane in (f).

SI 4A. Simulation experiments: Comparing Jump-Switch-Flow and Doob-Gillespie

We compare our Jump-Switch-Flow method to the gold standard approach for simulating exact solutions to CTMCs, the Doob-Gillespie method. To do so, we first specified an initial population size of $N(0) = 10^5$, and a switching threshold of each compartment of $\Omega = 10^3$. We then generated 5,000 simulations using the parameters in Table SI 2.

To capture the dynamics of the model, we track the infectious compartment as a key compartment of interest. In doing so, we then obtain distributions for the peak number of infectious individuals and also the time for the infection to peak, for the fade-out scenario. We also track the cumulative number of infected individuals after a certain amount of time for the endemic scenario. These results are presented in Figure SI 9.

Comparing Figures SI 8a and SI 8f, we can immediately observe that the distribution for time to peak for generated by the Jump-Switch-Flow method is similar to that of the Doob-Gillespie method. This is because the delay in the epidemic take off at low population is fully captured with this sufficiently large choice of switching threshold, $\Omega = 10^3$, enabling the full stochastic effects to be accurately represented.

However, if we compare the distributions for the peak number of infectious individuals (SI 8c and SI 8h), we can see that they differ significantly, for this chosen switching threshold. This difference can be understood if we consider how the process is behaving. Once the infectious compartment is sufficiently large for the Jump-Switch-Flow method, the method switches from a stochastic representation to a deterministic representation. Therefore, once the infectious compartment becomes deterministic, given that the susceptible population is also deterministic, its peak value is determined by the number of both infectious and susceptible individuals at this time. Since the number of infectious people at this time will always be the same, $I = \Omega = 10^3$ people, any variation in peak value is due to the number of susceptible individuals, S, at the time of switching.

If the summary statistic we instead compare is the distribution of the cumulative number of infected individuals after four years of epidemic circulation, we observe that, even for a relatively low switching threshold, the Jump-Switch-Flow method performs well comparatively to the Doob-Gillespie method, see Figures SI 8e and SI 8j. We also note that for these parameter choices and switching threshold, the infectious compartment switches between jumping and flowing states multiple times, yet the Jump-Switch-Flow method produces a cumulative distribution comparable to that of Doob-Gillespie. This indicates that the Jump-Switch-Flow method is robust for long term simulations.

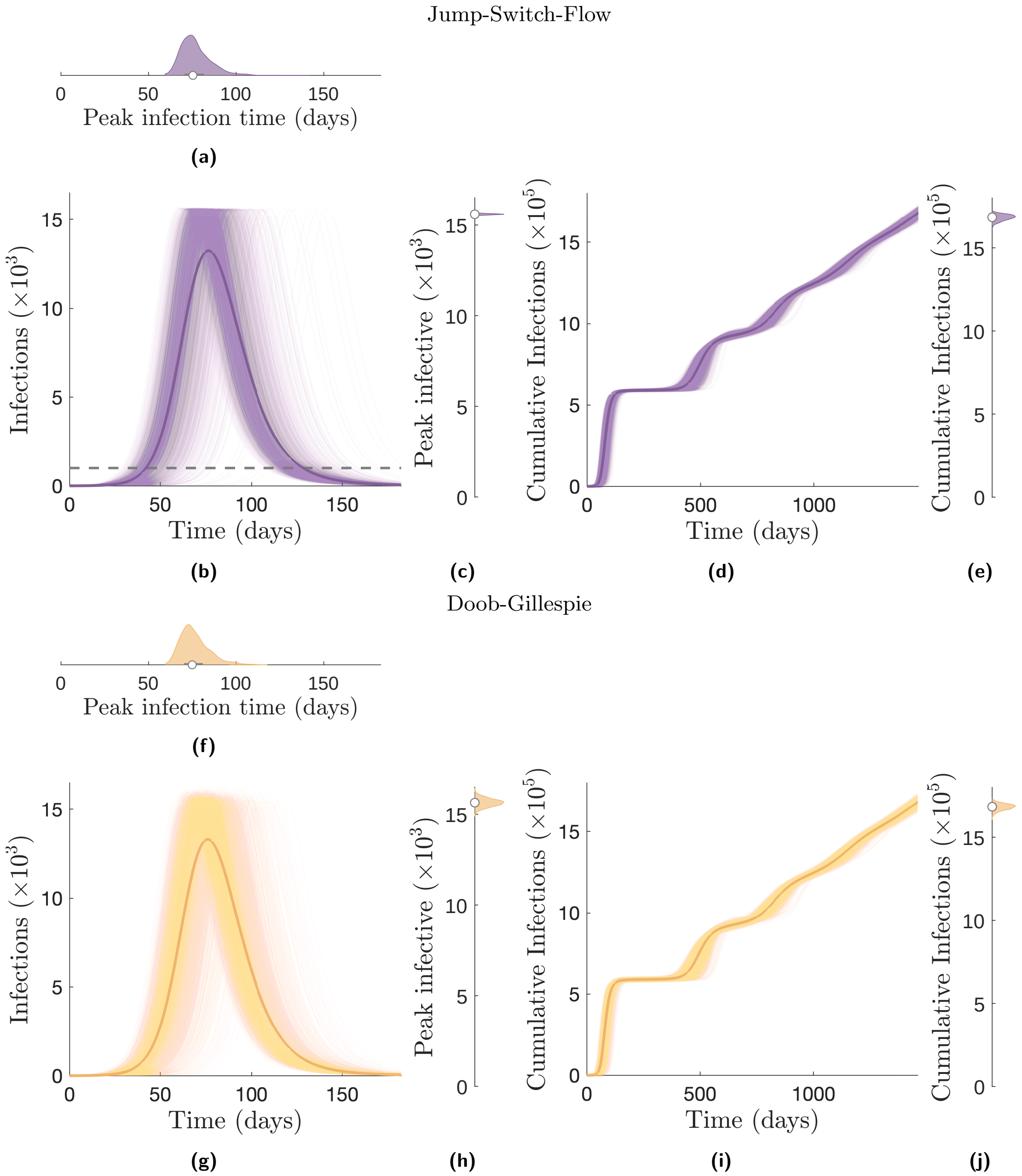

Figure SI 8: We compare the infectious compartment from realisations for Jump-Switch-Flow (b) and Doob-Gillespie (g) solutions, for a population size of $N(0) = 10^5$. We use a switching threshold of $\Omega = 10^3$. We also show the distributions of the time to infectious peak ((a) and (f)), and infectious peak values ((c) and (h)). We compare the cumulative infections over the simulations for Jump-Switch-Flow (d) and Doob-Gillespie (i) solutions and the final distributions ((e) and (j)). Point averages are also presented as a solid line.

The final summary statistics we are interested in are the probabilities of extinction, fade-out and endemic scenarios. Table SI 3 shows the probability of each scenario computed from 5,000 simulated samples. Here, we see that both methods produce comparatively similar results, which indicates that the Jump-Switch-Flow method is capable of capturing the inherent stochasticity in the exact simulation of the system via the Doob-Gillespie method.

Method	Extinction	Fade-out	Endemic
Jump-Switch-Flow	0.2476 ± 0.0120	0.4774 ± 0.0138	0.2750 ± 0.0124
Doob-Gillespie	0.2520 ± 0.0120	0.4668 ± 0.0138	0.2812 ± 0.0125

Table SI 3: Probabilities (with associated 95% confidence interval) of scenario outcomes for Jump-Switch-Flow and Doob-Gillespie, from 5,000 simulations.

We also compare the Jump-Switch-Flow and Doob-Gillespie methods where the switching threshold has been set to the population size, resulting in no compartments switching into the flowing state. These results are further discussed in SI 4B, where we show the

two methods are indistinguishable.

SI 4B. Simulation experiments: Comparing Jump-Switch-Flow (with no switching) and Doob-Gillespie

We compare the Jump-Switch-Flow method to the Doob-Gillespie method. To implement the latter, we set the switching threshold to be the total population size, to ensure no compartments switch into a flowing regime, and therefore all compartments are completely stochastic. We can therefore investigate how our jumping process compares to exact solutions of the continuous-time Markov chains.

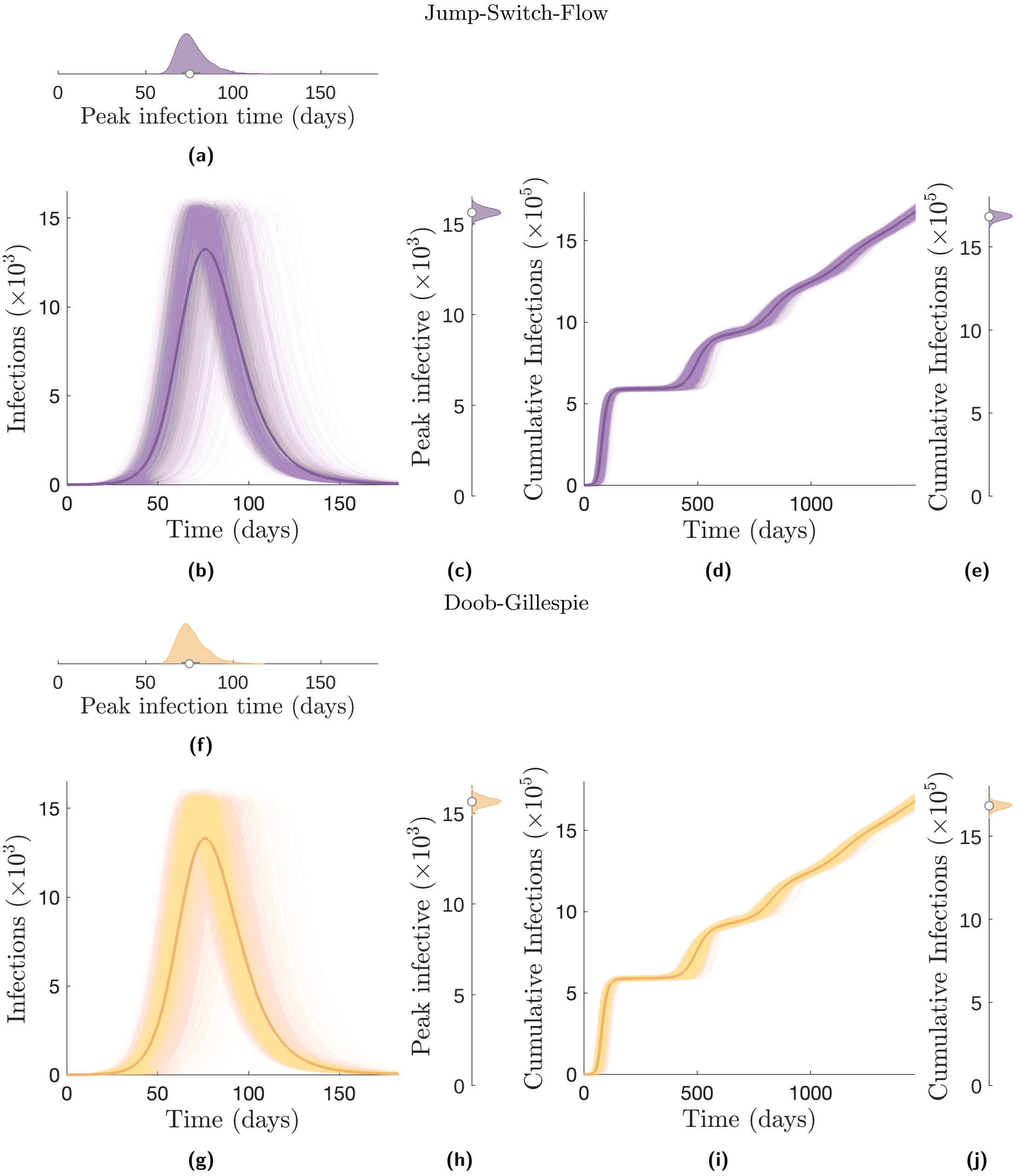

Figure SI 9: We compare the infectious compartment from realisations for Jump-Switch-Flow (b) and Doob-Gillespie (g) solutions, for a population size of $N_0 = 10^5$. We use a switching threshold of $\Omega = 10^3$. We also show the distributions of the time to infectious peak ((a) and (f)), and infectious peak values ((c) and (h)). We compare the cumulative infections over the simulations for Jump-Switch-Flow (d) and Doob-Gillespie (i) solutions and the final distributions ((e) and (j)). Point averages are also presented as a solid line.

Using parameter values in Table 3 of the main text, we generated 5,000 simulations, tracking the same quantities of interest. As with the case with compartment switching, the peak infection time distribution of JSF (Figure SI 9a) and Doob-Gillespie (Figure SI 9f) are comparable. However, this time, as well as a similar median peak infective individuals, the distributions of both JSF (Figure SI 9c)

and Doob-Gillespie (Figure SI 9h) are also comparable. Lastly, as we saw with compartment switching, the distributions of cumulative infections after four years are comparable for both JSF (Figure SI 9e) and Doob-Gillespie (Figure SI 9j).

Table SI 4 shows that the probabilities of scenario outcomes are also comparable for both Jump-Switch-Flow (with no switching) and Doob-Gillespie.

Method	Extinction	Fade-out	Endemic
Jump-Switch-Flow	0.2512 ± 0.0120	0.4700 ± 0.0139	0.2788 ± 0.0123
Doob-Gillespie	0.2520 ± 0.0120	0.4668 ± 0.0139	0.2812 ± 0.0125

Table SI 4: Probabilities (with associated 95% confidence interval) of scenario outcomes for Jump-Switch-Flow (with no switching) and Doob-Gillespie, from 5,000 simulations.

SI 4C. Simulation experiments: Sensitivity of Jump-Switch-Flow to switching threshold

In this section, we demonstrate how the accuracy of the Jump-Switch-Flow method can exhibit sensitivity with respect to the switching threshold (Figure SI 10). In Figure SI 10a, we can see that the distribution of peak number of infective individuals for the fade-out scenario is highly sensitive to the switching threshold. For a small switching threshold ($\Omega = 10^1$), we see that the distribution exhibits low variance, is representative of the deterministic expectation, and is not capturing the distribution obtained with the Doob-Gillespie method. As the switching threshold is increased, we see that the distribution of the peak number of infective individuals increases in variance. However, the Jump-Switch-Flow method is not representative of the Doob-Gillespie method until a large switching threshold ($\Omega = N(0) = 10^5$), where all compartments are in the jumping state.

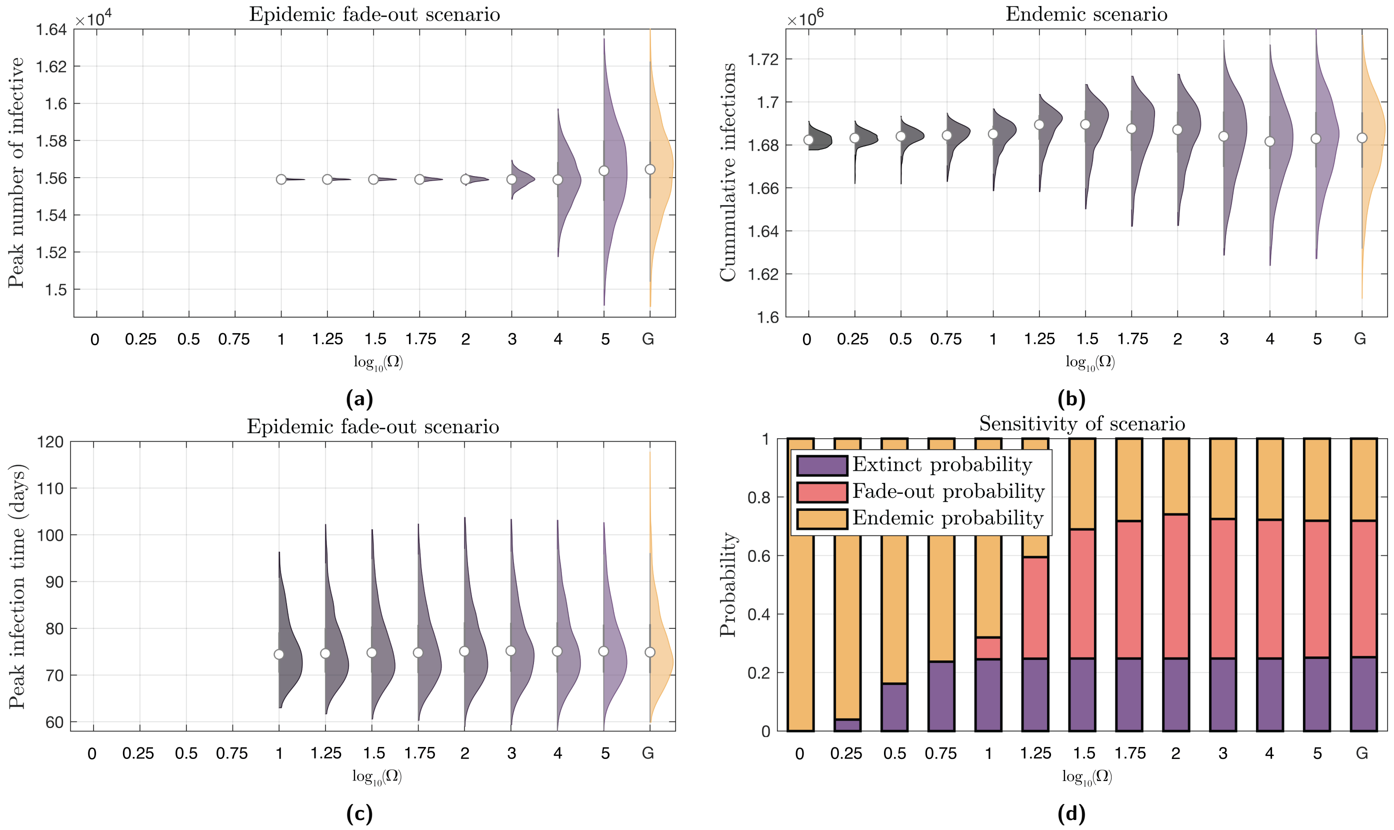

Figure SI 10: Summary statistics of interest to observe the sensitivity of the Jump-Switch-Flow method with respect to the switching threshold. (a) Sensitivity to the distribution of peak number of infective individuals for the fade-out scenario. (c) Sensitivity to the distribution of peak infection time for the fade-out scenario. (b) Sensitivity to the distribution of the cumulative number of infected individuals after four years for the endemic scenario. (d) Sensitivity to the probability of each scenario occurring. In each of the plots (a)-(c) grey to purple shaded distributions represent results obtained with the Jump-Switch-Flow method whilst the yellow distributions are the gold standard Doob-Gillespie algorithm (for which the placeholder 'G' is used to signify) which exhibit exact stochasticity but at the cost of high computational requirements.

Figure SI 10c presents the distribution of peak infection time for the fade-out scenario for various switching thresholds, and comparing

to the Doob-Gillespie method. Here, we see that the Jump-Switch-Flow method with a relatively small switching threshold ($\Omega = 10^2$) is capable of capturing a representative distribution of the Doob-Gillespie method.

Similarly, Figure SI 10b presents the distribution of the cumulative number of infected individuals after four years for the endemic scenario. Here, we again see that the Jump-Switch-Flow method with a relatively small switching threshold ($\Omega = 10^3$) is also representative of the Doob-Gillespie method.

Lastly, Figure SI 10d shows the probability of each scenario occurring (extinction, fade-out, endemic). Here, we see that the Jump-Switch-Flow method with a switching threshold of $\Omega \leq 10^1$ over-represents the endemic scenario. However, increasing the switching threshold, we observe that the probability of each scenario occurring quickly becomes representative of those found by the Doob-Gillespie method.

SI 4D. Simulation experiments: Computational efficiency of Jump-Switch-Flow

We now present the computational efficiency of the Jump-Switch-Flow method, and compare it to the Doob-Gillespie method and Tau-Leaping method (which is regarded as a computationally efficient, approximate, method), and the Tau-hybrid method, provided by GillesPy2 [3]. We implement two variants of Tau-leaping with a fixed step size of $\Delta\tau = 0.01$ days, and $\Delta\tau = 0.1$ days respectively, two variants of the C++ implementation of the Tau-hybrid method, with time steps of size $\Delta t = 0.01$ days and $\Delta t = 0.1$ days respectively, one implementation of the Python Tau-hybrid method, with time steps of size $\Delta t = 0.1$ days, and the Jump-Switch-Flow method with a time step size of $\Delta t = 0.01$ days.

Figure SI 11a shows how the computational efficiency varies with the switching thresholds, Ω, for the SIRS model with demography presented in the previous section, and compares it to the Doob-Gillespie method. We see that as we increase the switching threshold, the Jump-Switch-Flow method requires more computational time to simulate the scenario for four years, but is always more efficient than the Doob-Gillespie method. This increased efficiency is because the Jump-Switch-Flow method utilises the Next Reaction Method to compute the stochastic events, a method known to be more computationally efficient than the direct, first reaction, Doob-Gillespie method.

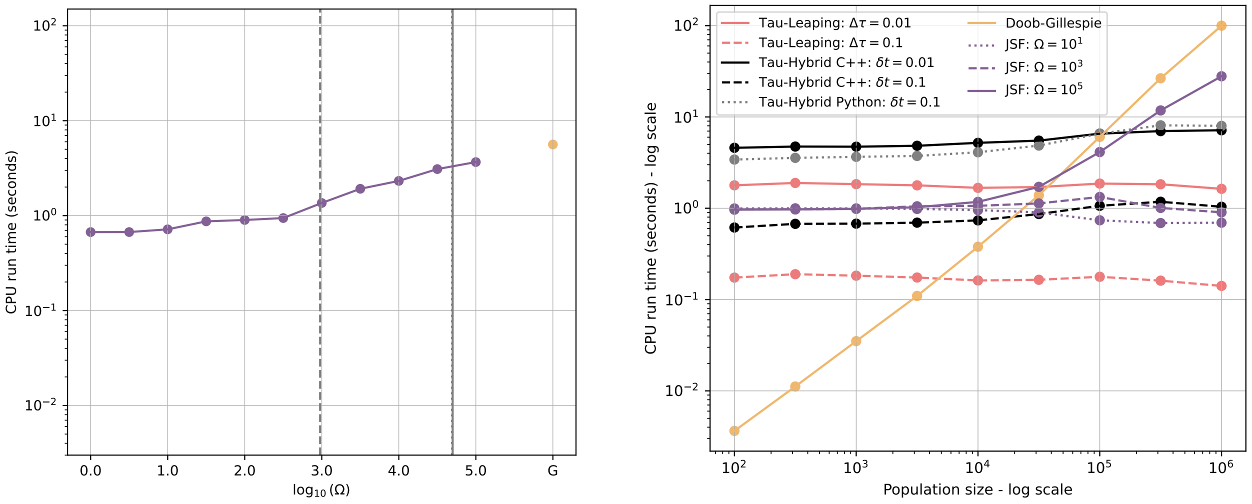

(a) (b)

Figure SI 11: (a) The running time to simulate the scenario for four years for an initial population size of $N(0) = 10^5$, with varying switching threshold, Ω (grey to purple), compared with the Doob-Gillespie ('G') method (yellow). The endemic steady state number of infectious individuals (vertical dashed line), susceptible individuals (vertical solid line), and recovered individuals (vertical dotted line) are presented. (b) The running time for the Jump-Switch-Flow method for an appropriate threshold choice is approximately constant as the population size varies (for example $\Omega = 10^3$). If a poor switching threshold is chosen, the running time begins to scale exponentially (for example $\Omega = 10^5$). For the Tau-Leaping method, the running time is constant, as we use fixed step size of $\Delta\tau = 0.01$ (solid line) and $\Delta\tau = 0.1$ (dashed line). We also present the Tau-hybrid method, in both Python and C++, with various different time meshes, C++ with a fine mesh ($\delta t = 0.01$, black solid line) and a corse mesh ($\delta t = 0.1$, black dashed line), and Python with a corse mesh ($\delta t = 0.1$, grey dotted line).

To observe how the computational efficiency of our Jump-Switch-Flow method varies with the total (population) size of the system, we consider three switching thresholds: $\Omega = 10^1$ (dotted purple), $\Omega = 10^3$ (dashed purple), and $\Omega = 10^5$ (solid purple). Figure SI 11b compares the Jump-Switch-Flow method (purple) to the Doob-Gillespie method (yellow), to the computationally more efficient

approximation Tau-Leaping method ($\Delta\tau = 0.01$, dashed red, and $\Delta\tau = 0.1$, solid red), and to the Tau-hybrid method ($\Delta t = 0.01$ solid black, and $\Delta t = 0.1$ dashed black) provided by GillesPy2, noting the log-log scale. We simulate the SIRS system described above, for a total time of 4×365 days. We can see that the computational efficiency of the Doob-Gillespie method scales exponentially with system size, as expected. The Tau-Leaping method initially has constant computational time scaling. In contrast, our Jump-Switch-Flow method is constant in time up to an initial system size of $N(0) = 10^5$, after which it begins to slowly decrease (for $\Omega = 10^1$ and $\Omega = 10^3$). This decrease is caused by more of the scenario being simulated in the flowing state, as the system becomes highly deterministic with a larger population. We also observe our Jump-Switch-Flow method increasing exponentially between $N(0) = 10^4$, for the largest switching threshold of $\Omega = 10^5$ (solid purple). We observe our Jump-Switch-Flow method performing better, in comparison to Doob-Gillespie exact method, as we use the Next Reaction Method to sample the Jump times. This is despite our implementation being required to perform extra computation to check the regime of the Jump-Switch-Flow process, and implement any relevant regime changes (even though they are not required in this particular instance). We also observe that for large, realistic population sizes (i.e. $N(0) \geq 10^5$), the Tau-hybrid method with the corse time mesh (black dashed line) performs at best comparable to, or worse than the Jump-Switch-Flow method, if a suitable Ω is chosen (such as $\Omega = 10^3$). Moreover, as we specify a finer time mesh (solid black line) we observe that the Tau-hybrid method performs worse across all population sizes. We anticipate this is due to the Tau-hybrid method utilising an arbitrary ODE solver, an adaptive $\Delta\tau$ that is calculated at each step, and also due to the dynamic switching behaviour between stochastic and deterministic regimes that requires extra computation of the propensities to determine if a switching event should occur.

SI 4E. Simulation experiments: Inference with simulated data, parameter estimates

To test how the Jump-Switch-Flow method behaves when used to perform parameter estimation, we first generated synthetic data of a known SIRS mode with demography. We simulate data for 400 days using the parameter values given in Figure 2C, with the Doob-Gillespie algorithm, and select a trajectory which experiences epidemic fade-out (at day 250), and an initial condition of two infected individuals ($I(0) = 2$), no recovered individuals ($R(0) = 0$), and a total population of $N(0) = 10^5$ individuals. We track the number of infectious individuals within the population each day, and use a truncated binomial distribution to add noise into the data set, reflecting a realistic measurement process. We then couple our Jump-Switch-Flow method, using a switching threshold of $\Omega = 10^3$, with the open-source particle filter package pypfilt [4], using 2,000 particles. We first fix the demographic parameters to $\kappa = \mu = 1/(85 \times 365)$ (since these are assumed to be standard within a given population). We perform the inference on the data set for the first 100 days of the epidemic to estimate β, γ and ω, and then use our parameter estimates to predict forward in time the possible trajectories based on the obtained predictions. Using these predictions, we also estimate the probability of epidemic fade-out occurring at times $t = 100$ to $t = 400$ days.

Figure SI 12 shows the posterior distributions of parameters. The dotted black lines show the true value used to produce the simulated data, and the dashed red lines indicate the initial bounds on the prior estimates. We see that both β and γ are well estimated, with the posteriors centred around the true values. However, the posterior marginal of ω is less concentrated on the true value. This is because ω acts on the order of 1 year, while the window of data used to fit the parameters to the data is 100 days (≈ 0.3 years).

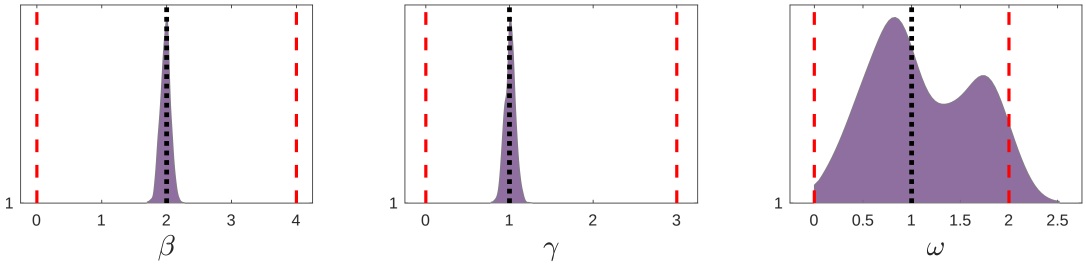

Figure SI 12: Posterior distributions of parameters for the SIRS model with demography, obtained via a particle filter with 2,000 particles, and the Jump-Switch-Flow sampler. The red dashed lines indicate the initial bounds on prior samples. The vertical dotted black lines indicate the true value used to produce the simulated data.

SI 5. Inference Study: Estimating TEIRV model parameters using a particle filter

We implemented the Refractory Cell Model described by the authors for the viral load data obtained from nasal swabs, using our Jump-Switch-Flow method with a switching threshold on all compartments $\Omega = 10^2$. We implemented this in particle filter pypfilt [4] to estimate the model parameters, using 6,000 particles per simulation. In previous analysis [2], the decay of virion was fixed at $c = 10$ virions/day, and the eclipse of cells to become infectious as $k = 4$ cells/day, leaving the remaining parameters to be estimated. Therefore, we similarly fix $c = 10$ virions/day and $k = 4$ cells/day. The prior distributions used for the analysis with the TEIRV model are given in Table SI 5.

Parameter	Prior
$\log_e(V_0)$	Uniform$(0, 5)$
c	Fixed at 10.0
k	Fixed at 4.0
$\beta \times 10^7$	Uniform$(0, 20)$
$\Phi \times 10^5$	Uniform$(0, 15)$
ρ	Uniform$(0, 1)$
δ	Uniform$(1, 10)$
π	Uniform$(200, 400)$

Table SI 5: The prior distributions used for the analysis with the TEIRV model.

SI 5A. Parameter Estimation and Viral Clearance Prediction

Figure 9 of the main text highlights how the estimated viral loads closely resemble the data collected from the 6 patients. We also show how the estimated R_0 values for the patients vary. These R_0 estimates are consistent with the original study by [2]. However, understanding how the parameter estimates vary between patients assists in understanding how the fitting process behaves.

The posterior distributions for each of the 6 patient's parameter estimates are provided in Figure SI 13. These posterior distributions are found by fitting a violin envelope from the 6,000 estimates obtained from the particle filtering process. We also show the prior distributions, as red dashed lines, which show the lower and upper bounds for the uniform initial guesses.

We can see that, often, parameters ρ and Φ are not identified here, as the posterior distributions closely resemble the priors (excluding patient 451152). We also observed that for all patients, β appears to be well estimated. The parameter π is also well estimated for patients 443108, 444332, 444391 and 451152, however patients 432192 and 445602 provide less insight. The parameter δ is very well estimated by all patients, excluding patient 451152. Lastly, the initial viral loads, $\log_{10}(V_0)$, are all estimated well, and also estimated to take a large value.

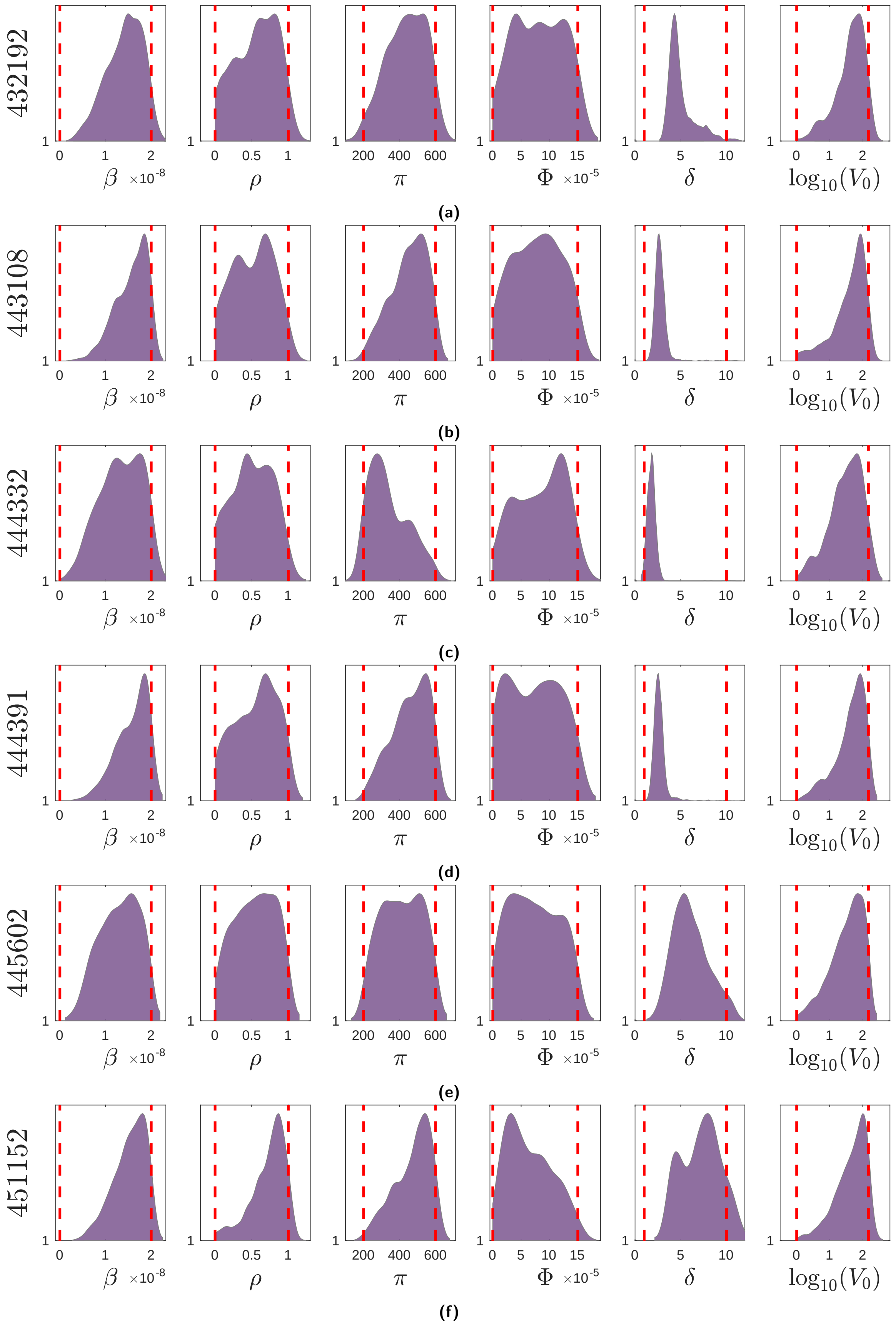

Figure SI 13: Posterior distributions of the model parameters and initial viral load for each of the selected SARS-CoV-2 infections. The red dashed lines indicate the initial (uniform) bounds on prior samples.
